# Effectiveness of weight management interventions for adults delivered in primary care: systematic review and meta-analysis of randomised controlled trials

**DOI:** 10.1136/bmj-2021-069719

**Published:** 2022-05-31

**Authors:** Claire D Madigan, Henrietta E Graham, Elizabeth Sturgiss, Victoria E Kettle, Kajal Gokal, Greg Biddle, Gemma M J Taylor, Amanda J Daley

**Affiliations:** 1 Centre for Lifestyle Medicine and Behaviour (CLiMB), The School of Sport, Exercise and Health Sciences, Loughborough University, Loughborough LE11 3TU, UK; 2 School of Primary and Allied Health Care, Monash University, Melbourne, Australia; 3 Department of Psychology, Addiction and Mental Health Group, University of Bath, Bath, UK

## Abstract

**Objective:**

To examine the effectiveness of behavioural weight management interventions for adults with obesity delivered in primary care.

**Design:**

Systematic review and meta-analysis of randomised controlled trials.

**Eligibility criteria for selection of studies:**

Randomised controlled trials of behavioural weight management interventions for adults with a body mass index ≥25 delivered in primary care compared with no treatment, attention control, or minimal intervention and weight change at ≥12 months follow-up.

**Data sources:**

Trials from a previous systematic review were extracted and the search completed using the Cochrane Central Register of Controlled Trials, Medline, PubMed, and PsychINFO from 1 January 2018 to 19 August 2021.

**Data extraction and synthesis:**

Two reviewers independently identified eligible studies, extracted data, and assessed risk of bias using the Cochrane risk of bias tool. Meta-analyses were conducted with random effects models, and a pooled mean difference for both weight (kg) and waist circumference (cm) were calculated.

**Main outcome measures:**

Primary outcome was weight change from baseline to 12 months. Secondary outcome was weight change from baseline to ≥24 months. Change in waist circumference was assessed at 12 months.

**Results:**

34 trials were included: 14 were additional, from a previous review. 27 trials (n=8000) were included in the primary outcome of weight change at 12 month follow-up. The mean difference between the intervention and comparator groups at 12 months was −2.3 kg (95% confidence interval −3.0 to −1.6 kg, I^2^=88%, P<0.001), favouring the intervention group. At ≥24 months (13 trials, n=5011) the mean difference in weight change was −1.8 kg (−2.8 to −0.8 kg, I^2^=88%, P<0.001) favouring the intervention. The mean difference in waist circumference (18 trials, n=5288) was −2.5 cm (−3.2 to −1.8 cm, I^2^=69%, P<0.001) in favour of the intervention at 12 months.

**Conclusions:**

Behavioural weight management interventions for adults with obesity delivered in primary care are effective for weight loss and could be offered to members of the public.

**Systematic review registration:**

PROSPERO CRD42021275529.

## Introduction

Obesity is associated with an increased risk of diseases such as cancer, type 2 diabetes, and heart disease, leading to early mortality.^1^ ^2^ ^3^ More recently, obesity is a risk factor for worse outcomes with covid-19.^4^ ^5^ Because of this increased risk, health agencies and governments worldwide are focused on finding effective ways to help people lose weight.^6^

Primary care is an ideal setting for delivering weight management services, and international guidelines recommend that doctors should opportunistically screen and encourage patients to lose weight.^7^ ^8^ On average, most people consult a primary care doctor four times yearly, providing opportunities for weight management interventions.^9^ ^10^ A systematic review of randomised controlled trials by LeBlanc et al identified behavioural interventions that could potentially be delivered in primary care, or involved referral of patients by primary care professionals, were effective for weight loss at 12-18 months follow-up (−2.4 kg, 95% confidence interval −2.9 to−1.9 kg).^11^ However, this review included trials with interventions that the review authors considered directly transferrable to primary care, but not all interventions involved primary care practitioners. The review included interventions that were entirely delivered by university research employees, meaning implementation of these interventions might differ if offered in primary care, as has been the case in other implementation research of weight management interventions, where effects were smaller.^12^ As many similar trials have been published after this review, an updated review would be useful to guide health policy.

We examined the effectiveness of weight loss interventions delivered in primary care on measures of body composition (weight and waist circumference). We also identified characteristics of effective weight management programmes for policy makers to consider.

## Methods

This systematic review was registered on PROSPEROand is reported according to the preferred reporting items for systematic reviews and meta-analyses (PRISMA) statement.^13^ ^14^

### Eligibility criteria

We considered studies to be eligible for inclusion if they were randomised controlled trials, comprised adult participants (≥18 years), and evaluated behavioural weight management interventions delivered in primary care that focused on weight loss. A primary care setting was broadly defined as the first point of contact with the healthcare system, providing accessible, continued, comprehensive, and coordinated care, focused on long term health.^15^ Delivery in primary care was defined as the majority of the intervention being delivered by medical and non-medical clinicians within the primary care setting. Table 1 lists the inclusion and exclusion criteria.

**Table 1:** Study inclusion and exclusion criteria

	Inclusion criteria	Exclusion criteria
Study aim	Weight loss	Primary prevention of overweight or obesity; treatment of cardiovascular disease; treatment of cancer
Population	Adults aged ≥18 years who were candidates for weight loss interventions and were selected based on a body mass index (BMI) higher than normal range (≥25) or other weight related measure (eg, waist circumference)	Studies limited to populations not selected based on a weight related measure (ie, BMI, waist circumference, weight); adults with a known chronic disease not generalisable to the primary care population (eg, eating disorder, cancer, chronic kidney disease, severe mental disorder, cognitive impairment); children and adolescents; parents (if intended behaviour change was for offspring); pregnant women
Setting	Studies conducted in primary care and delivered by a staff member of the primary care health team within the practice (primary care was defined as the first point of contact, based in the community, and could offer ongoing and comprehensive healthcare)	
Interventions	Behavioural interventions focusing on weight loss; interventions could be delivered face to face or by telephone, print materials, or technology, and could be delivered individually or in groups. Interventions could be delivered by numerous potential healthcare professionals, including but not limited to doctors, nurses, exercise specialists, dietitians, nutritionists, health coaches, and behavioural health specialists; but they had to be employed by, and delivered within, primary care	Complementary and alternative treatments; surgical and drug treatment; dietary supplements intended for weight loss
Comparisons	No treatment (eg, wait list control, usual care); attention control (eg, similar format and intensity to intervention but different content area (eg, focusing on other behaviour)); minimal intervention comparable to usual care (including use of generic printed or electronic communications)	Active comparators without a control (as defined in inclusion criteria)
Outcomes	Weight outcomes: measured weight (eg, kilograms, pounds)	
Timing of outcome assessment	≥12 months after start of intervention or baseline assessment	

### Searches

We extracted studies from the systematic review by LeBlanc et al that met our inclusion criteria.^11^ We also searched the exclusions in this review because the researchers excluded interventions specifically for diabetes management, low quality trials, and only included studies from an Organisation for Economic Co-operation and Development country, limiting the scope of the findings.

We searched for studies in the Cochrane Central Register of Controlled Trials, Medline, PubMed, and PsychINFO from 1 January 2018 to 19 August 2021 (see supplementary file 1). Reference lists of previous reviews^16^ ^17^ ^18^ ^19^ ^20^ ^21^ and included trials were hand searched.

### Data extraction

Results were uploaded to Covidence,^22^ a software platform used for screening, and duplicates removed. Two independent reviewers screened study titles, abstracts, and full texts. Disagreements were discussed and resolved by a third reviewer. All decisions were recorded in Covidence, and reviewers were blinded to each other’s decisions. Covidence calculates proportionate agreement as a measure of inter-rater reliability, and data are reported separately by title or abstract screening and full text screening. One reviewer extracted data on study characteristics (see supplementary table 1) and two authors independently extracted data on weight outcomes. We contacted the authors of four included trials (from the updated search) for further information.^23^ ^24^ ^25^ ^26^

### Outcomes, summary measures, and synthesis of results

The primary outcome was weight change from baseline to 12 months. Secondary outcomes were weight change from baseline to ≥24 months and from baseline to last follow-up (to include as many trials as possible), and waist circumference from baseline to 12 months. Supplementary file 2 details the prespecified subgroup analysis that we were unable to complete. The prespecified subgroup analyses that could be completed were type of healthcare professional who delivered the intervention, country, intensity of the intervention, and risk of bias rating.

*Healthcare professional delivering intervention*—From the data we were able to compare subgroups by type of healthcare professional: nurses,^24^ ^26^ ^27^ ^28^ general practitioners,^23^ ^29^ ^30^ ^31^ and non-medical practitioners (eg, health coaches).^32^ ^33^ ^34^ ^35^ ^36^ ^37^ ^38^ ^39^ Some of the interventions delivered by non-medical practitioners were supported, but not predominantly delivered, by GPs. Other interventions were delivered by a combination of several different practitioners—for example, it was not possible to determine whether a nurse or dietitian delivered the intervention. In the subgroup analysis of practitioner delivery, we refer to this group as “other.”

*Country*—We explored the effectiveness of interventions by country. Only countries with three or more trials were included in subgroup analyses (United Kingdom, United States, and Spain).

*Intensity of interventions*—As the median number of contacts was 12, we categorised intervention groups according to whether ≤11 or ≥12 contacts were required.

*Risk of bias rating*—Studies were classified as being at low, unclear, and high risk of bias. Risk of bias was explored as a potential influence on the results.

### Meta-analyses

Meta-analyses were conducted using Review Manager 5.4.^40^ As we expected the treatment effects to differ because of the diversity of intervention components and comparator conditions, we used random effects models. A pooled mean difference was calculated for each analysis, and variance in heterogeneity between studies was compared using the I^2^ and τ^2^ statistics. We generated funnel plots to evaluate small study effects. If more than two intervention groups existed, we divided the number of participants in the comparator group by the number of intervention groups and analysed each individually. Nine trials were cluster randomised controlled trials. The trials had adjusted their results for clustering, or adjustment had been made in the previous systematic review by LeBlanc et al.^11^ One trial did not report change in weight by group.^26^ We calculated the mean weight change and standard deviation using a standard formula, which imputes a correlation for the baseline and follow-up weights.^41^ ^42^ In a non-prespecified analysis, we conducted univariate and multivariable metaregression (in Stata) using a random effects model to examine the association between number of sessions and type of interventionalist on study effect estimates.

### Risk of bias

Two authors independently assessed the risk of bias using the Cochrane risk of bias tool v2.^43^ For incomplete outcome data we defined a high risk of bias as ≥20% attrition. Disagreements were resolved by discussion or consultation with a third author.

### Patient and public involvement

The study idea was discussed with patients and members of the public. They were not, however, included in discussions about the design or conduct of the study.

## Results

**Fig 1:**
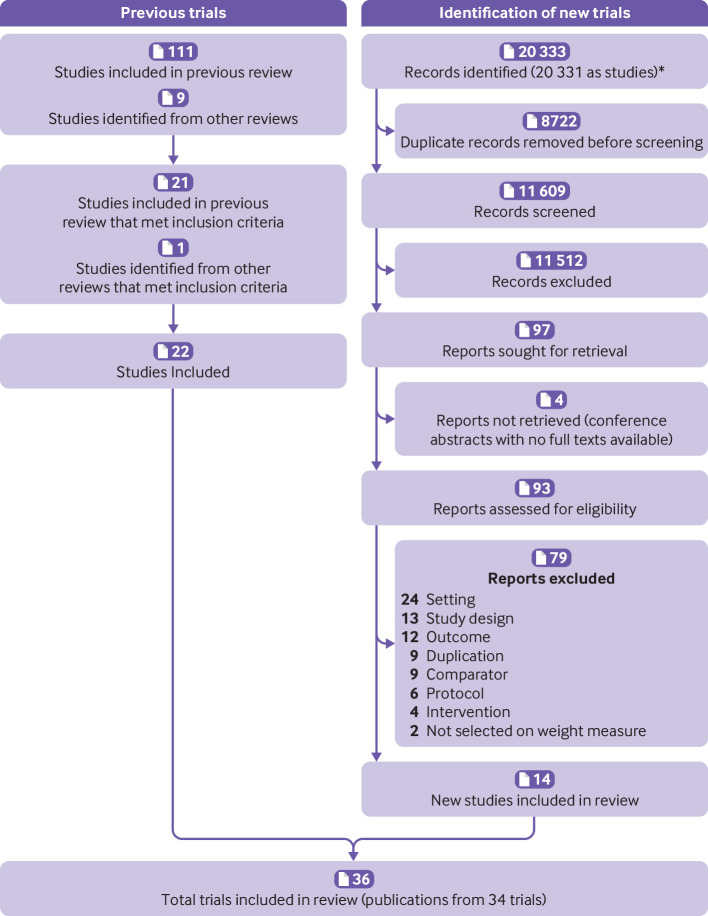
Studies included in systematic review of effectiveness of behavioural weight management interventions in primary care. *Studies were merged in Covidence if they were from same trial

The search identified 11 609 unique study titles or abstracts after duplicates were removed (fig 1). After screening, 97 full text articles were assessed for eligibility. The proportionate agreement ranged from 0.94 to 1.0 for screening of titles or abstracts and was 0.84 for full text screening. Fourteen new trials met the inclusion criteria. Twenty one studies from the review by LeBlanc et al met our eligibility criteria and one study from another systematic review was considered eligible and included.^44^ Some studies had follow-up studies (ie, two publications) that were found in both the second and the first search; hence the total number of trials was 34 and not 36. Of the 34 trials, 27 (n=8000 participants) were included in the primary outcome meta-analysis of weight change from baseline to 12 months, 13 (n=5011) in the secondary outcome from baseline to ≥24 months, and 30 (n=8938) in the secondary outcome for weight change from baseline to last follow-up. Baseline weight was accounted for in 18 of these trials, but in 14^24^ ^26^ ^29^ ^30^ ^31^ ^32^ ^44^ ^45^ ^46^ ^47^ ^48^ ^49^ ^50^ ^51^ it was unclear or the trials did not consider baseline weight. Eighteen trials (n=5288) were included in the analysis of change in waist circumference at 12 months.

### Study characteristics

Included trials (see supplementary table 1) were individual randomised controlled trials (n=25)^24^ ^25^ ^26^ ^27^ ^28^ ^29^ ^32^ ^33^ ^34^ ^35^ ^38^ ^39^ ^41^ ^44^ ^45^ ^46^ ^47^ ^50^ ^51^ ^52^ ^53^ ^54^ ^55^ ^56^ ^59^ or cluster randomised controlled trials (n=9).^23^ ^30^ ^31^ ^36^ ^37^ ^48^ ^49^ ^57^ ^58^ Most were conducted in the US (n=14),^29^ ^30^ ^31^ ^32^ ^33^ ^34^ ^35^ ^36^ ^37^ ^45^ ^48^ ^51^ ^54^ ^55^ UK (n=7),^27^ ^28^ ^38^ ^41^ ^47^ ^57^ ^58^ and Spain (n=4).^25^ ^44^ ^46^ ^49^ The median number of participants was 276 (range 50-864).

Four trials included only women (average 65.9% of women).^31^ ^48^ ^51^ ^59^ The mean BMI at baseline was 35.2 (SD 4.2) and mean age was 48 (SD 9.7) years. The interventions lasted between one session (with participants subsequently following the programme unassisted for three months) and several sessions over three years (median 12 months). The follow-up period ranged from 12 months to three years (median 12 months). Most trials excluded participants who had lost weight in the past six months and were taking drugs that affected weight.

### Meta-analysis

**Fig 2:**
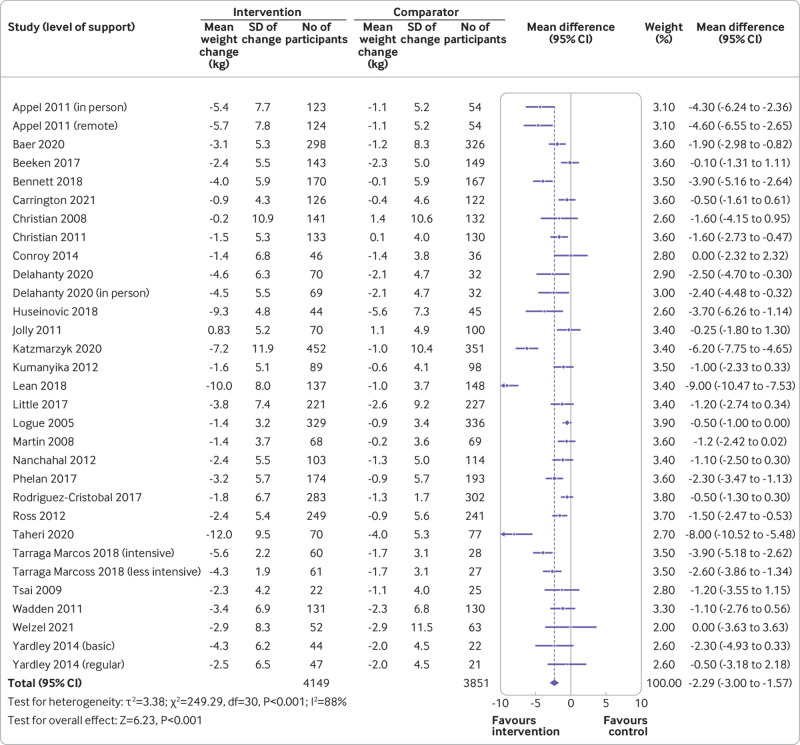
Mean difference in weight at 12 months by weight management programme in primary care (intervention) or no treatment, different content, or minimal intervention (control). SD=standard deviation

Overall, 27 trials were included in the primary meta-analysis of weight change from baseline to 12 months. Three trials could not be included in the primary analysis as data on weight were only available at two and three years and not 12 months follow-up, but we included these trials in the secondary analyses of last follow-up and ≥24 months follow-up.^26^ ^44^ ^50^ Four trials could not be included in the meta-analysis as they did not present data in a way that could be synthesised (ie, measures of dispersion).^25^ ^52^ ^53^ ^58^ The mean difference was −2.3 kg (95% confidence interval −3.0 to −1.6 kg, I^2^=88%, τ^2^=3.38; P<0.001) in favour of the intervention group (fig 2). We found no evidence of publication bias (see supplementary fig 1). Absolute weight change was −3.7 (SD 6.1) kg in the intervention group and −1.4 (SD 5.5) kg in the comparator group.

Supplementary file 2 provides a summary of the main subgroup analyses.

#### Weight change

The mean difference in weight change at the last follow-up was −1.9 kg (95% confidence interval −2.5 to −1.3 kg, I^2^=81%, τ^2^=2.15; P<0.001). Absolute weight change was −3.2 (SD 6.4) kg in the intervention group and −1.2 (SD 6.0) kg in the comparator group (see supplementary figs 2 and 3).

At the 24 month follow-up the mean difference in weight change was −1.8 kg (−2.8 to −0.8 kg, I^2^=88%, τ^2^=3.13; P<0.001) (see supplementary fig 4). As the weight change data did not differ between the last follow-up and ≥24 months, we used the weight data from the last follow-up in subgroup analyses.

In subgroup analyses of type of interventionalist, differences were significant (P=0.005) between non-medical practitioners, GPs, nurses, and other people who delivered interventions (see supplementary fig 2).

Participants who had ≥12 contacts during interventions lost significantly more weight than those with fewer contacts (see supplementary fig 6). The association remained after adjustment for type of interventionalist.

#### Waist circumference

**Fig 3:**
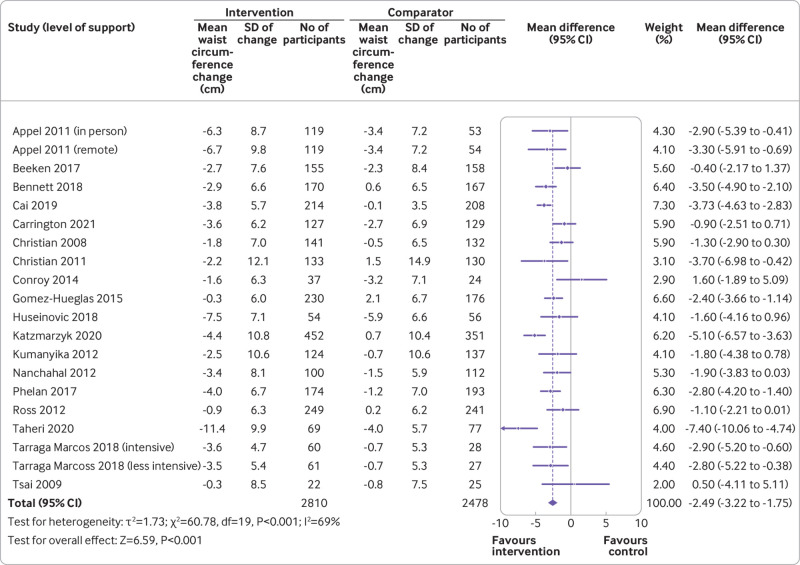
Mean difference in waist circumference at 12 months. SD=standard deviation

The mean difference in waist circumference was −2.5 cm (95% confidence interval −3.2 to −1.8 cm, I^2^=69%, τ^2^=1.73; P<0.001) in favour of the intervention at 12 months (fig 3). Absolute changes were −3.7 cm (SD 7.8 cm) in the intervention group and −1.3 cm (SD 7.3) in the comparator group.

#### Risk of bias

**Fig 4:**
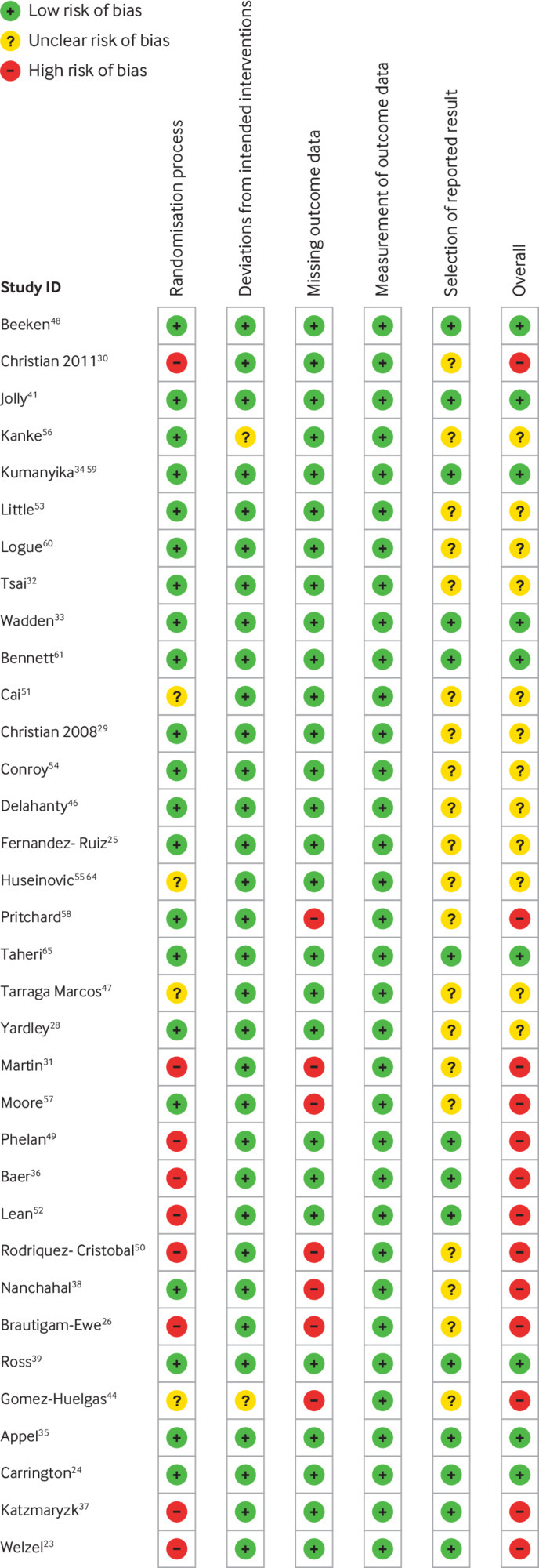
Risk of bias in included studies

Risk of bias was considered to be low in nine trials,^24^ ^33^ ^34^ ^35^ ^39^ ^41^ ^47^ ^55^ ^56^ unclear in 12 trials,^25^ ^27^ ^28^ ^29^ ^32^ ^45^ ^46^ ^50^ ^51^ ^52^ ^54^ ^59^ and high in 13 trials^23^ ^26^ ^30^ ^31^ ^36^ ^37^ ^38^ ^44^ ^48^ ^49^ ^53^ ^57^ ^58^ (fig 4). No significant (P=0.65) differences were found in subgroup analyses according to level of risk of bias from baseline to 12 months (see supplementary fig 7).

## Discussion

Worldwide, governments are trying to find the most effective services to help people lose weight to improve the health of populations. We found weight management interventions delivered by primary care practitioners result in effective weight loss and reduction in waist circumference and these interventions should be considered part of the services offered to help people manage their weight. A greater number of contacts between patients and healthcare professionals led to more weight loss, and interventions should be designed to include at least 12 contacts (face-to-face or by telephone, or both). Evidence suggests that interventions delivered by non-medical practitioners were as effective as those delivered by GPs (both showed statistically significant weight loss). It is also possible that more contacts were made with non-medical interventionalists, which might partially explain this result, although the metaregression analysis suggested the effect remained after adjustment for type of interventionalist. Because most comparator groups had fewer contacts than intervention groups, it is not known whether the effects of the interventions are related to contact with interventionalists or to the content of the intervention itself.

Although we did not determine the costs of the programme, it is likely that interventions delivered by non-medical practitioners would be cheaper than GP and nurse led programmes.^41^ Most of the interventions delivered by non-medical practitioners involved endorsement and supervision from GPs (ie, a recommendation or checking in to see how patients were progressing), and these should be considered when implementing these types of weight management interventions in primary care settings. Our findings suggest that a combination of practitioners would be most effective because GPs might not have the time for 12 consultations to support weight management.

Although the 2.3 kg greater weight loss in the intervention group may seem modest, just 2-5% in weight loss is associated with improvements in systolic blood pressure and glucose and triglyceride levels.^60^ The confidence intervals suggest a potential range of weight loss and that these interventions might not provide as much benefit to those with a higher BMI. Patients might not find an average weight loss of 3.7 kg attractive, as many would prefer to lose more weight; explaining to patients the benefits of small weight losses to health would be important.

### Strengths and limitations of this review

Our conclusions are based on a large sample of about 8000 participants, and 12 of these trials were published since 2018. It was occasionally difficult to distinguish who delivered the interventions and how they were implemented. We therefore made some assumptions at the screening stage about whether the interventionalists were primary care practitioners or if most of the interventions were delivered in primary care. These discussions were resolved by consensus. All included trials measured weight, and we excluded those that used self-reported data. Dropout rates are important in weight management interventions as those who do less well are less likely to be followed-up. We found that participants in trials with an attrition rate of 20% or more lost less weight and we are confident that those with high attrition rates have not inflated the results. Trials were mainly conducted in socially economic developed countries, so our findings might not be applicable to all countries. The meta-analyses showed statistically significant heterogeneity, and our prespecified subgroups analysis explained some, but not all, of the variance.

### Comparison with other studies

The mean difference of −2.3 kg in favour of the intervention group at 12 months is similar to the findings in the review by LeBlanc et al, who reported a reduction of −2.4 kg in participants who received a weight management intervention in a range of settings, including primary care, universities, and the community.^11^ ^61^ This is important because the review by LeBlanc et al included interventions that were not exclusively conducted in primary care or by primary care practitioners. Trials conducted in university or hospital settings are not typically representative of primary care populations and are often more intensive than trials conducted in primary care as a result of less constraints on time. Thus, our review provides encouraging findings for the implementation of weight management interventions delivered in primary care. The findings are of a similar magnitude to those found in a trial by Ahern et al that tested primary care referral to a commercial programme, with a difference of −2.7 kg (95% confidence interval −3.9 to −1.5 kg) reported at 12 month follow-up.^62^ The trial by Ahern et al also found a difference in waist circumference of −4.1 cm (95% confidence interval −5.5 to −2.3 cm) in favour of the intervention group at 12 months. Our finding was smaller at −2.5 cm (95% confidence interval −3.2 to −1.8 cm). Some evidence suggests clinical benefits from a reduction of 3 cm in waist circumference, particularly in decreased glucose levels, and the intervention groups showed a 3.7 cm absolute change in waist circumference.^63^

### Policy implications and conclusions

Weight management interventions delivered in primary care are effective and should be part of services offered to members of the public to help them manage weight. As about 39% of the world’s population is living with obesity, helping people to manage their weight is an enormous task.^64^ Primary care offers good reach into the community as the first point of contact in the healthcare system and the remit to provide whole person care across the life course.^65^ When developing weight management interventions, it is important to reflect on resource availability within primary care settings to ensure patients’ needs can be met within existing healthcare systems.^66^

We did not examine the equity of interventions, but primary care interventions may offer an additional service and potentially help those who would not attend a programme delivered outside of primary care. Interventions should consist of 12 or more contacts, and these findings are based on a mixture of telephone and face-to-face sessions. Previous evidence suggests that GPs find it difficult to raise the issue of weight with patients and are pessimistic about the success of weight loss interventions.^67^ Therefore, interventions should be implemented with appropriate training for primary care practitioners so that they feel confident about helping patients to manage their weight.^68^

### Unanswered questions and future research

A range of effective interventions are available in primary care settings to help people manage their weight, but we found substantial heterogeneity. It was beyond the scope of this systematic review to examine the specific components of the interventions that may be associated with greater weight loss, but this could be investigated by future research. We do not know whether these interventions are universally suitable and will decrease or increase health inequalities. As the data are most likely collected in trials, an individual patient meta-analysis is now needed to explore characteristics or factors that might explain the variance. Most of the interventions excluded people prescribed drugs that affect weight gain, such as antipsychotics, glucocorticoids, and some antidepressants. This population might benefit from help with managing their weight owing to the side effects of these drug classes on weight gain, although we do not know whether the weight management interventions we investigated would be effective in this population.^69^

**What is already known on this topic**

Referral by primary care to behavioural weight management programmes is effective, but the effectiveness of weight management interventions delivered by primary care is not known
Systematic reviews have provided evidence for weight management interventions, but the latest review of primary care delivered interventions was published in 2014
Factors such as intensity and delivery mechanisms have not been investigated and could influence the effectiveness of weight management interventions delivered by primary care

**What this study adds**

Weight management interventions delivered by primary care are effective and can help patients to better manage their weight
At least 12 contacts (telephone or face to face) are needed to deliver weight management programmes in primary care
Some evidence suggests that weight loss after weight management interventions delivered by non-medical practitioners in primary care (often endorsed and supervised by doctors) is similar to that delivered by clinician led programmes

## Data Availability

Additional data are available in the supplementary files.

## Web extra

Extra material supplied by authors

## Notes

Contributors: CDM and AJD conceived the study, with support from ES. CDM conducted the search with support from HEG. CDM, AJD, ES, HEG, KG, GB, and VEK completed the screening and full text identification. CDM and VEK completed the risk of bias assessment. CDM extracted data for the primary outcome and study characteristics. HEJ, GB, and KG extracted primary outcome data. CDM completed the analysis in RevMan, and GMJT completed the metaregression analysis in Stata. CDM drafted the paper with AJD. All authors provided comments on the paper. CDM acts as guarantor. The corresponding author attests that all listed authors meet authorship criteria and that no others meeting the criteria have been omitted.

The lead author (CDM) affirms that the manuscript is an honest, accurate, and transparent account of the study being reported, and that no important aspects of the study have been omitted.

Dissemination to participants and related patient and public communities: We plan to disseminate these research findings to a wider community through press releases, featuring on the Centre for Lifestyle Medicine and Behaviour website (www.lboro.ac.uk/research/climb/) via our policy networks, through social media platforms, and presentation at conferences.

Provenance and peer review: Not commissioned; externally peer reviewed.

## References

[1] Renehan AG Tyson M Egger M Heller RF Zwahlen M . Body-mass index and incidence of cancer: a systematic review and meta-analysis of prospective observational studies. Lancet 2008;371:569-78. 10.1016/S0140-6736(08)60269-X 18280327

[2] Guh DP Zhang W Bansback N Amarsi Z Birmingham CL Anis AH . The incidence of co-morbidities related to obesity and overweight: a systematic review and meta-analysis. BMC Public Health 2009;9:88. 10.1186/1471-2458-9-88 19320986 PMC2667420

[3] Abdullah A Peeters A de Courten M Stoelwinder J . The magnitude of association between overweight and obesity and the risk of diabetes: a meta-analysis of prospective cohort studies. Diabetes Res Clin Pract 2010;89:309-19. 10.1016/j.diabres.2010.04.012. 20493574

[4] Aghili SMM Ebrahimpur M Arjmand B . Obesity in COVID-19 era, implications for mechanisms, comorbidities, and prognosis: a review and meta-analysis. Int J Obes (Lond) 2021;45:998-1016. 10.1038/s41366-021-00776-8. 33637951 PMC7909378

[5] Földi M Farkas N Kiss S KETLAK Study Group . Obesity is a risk factor for developing critical condition in COVID-19 patients: A systematic review and meta-analysis. Obes Rev 2020;21:e13095. 10.1111/obr.13095. 32686331 PMC7404429

[6] Department of Health and Social Care. New specialised support to help those living with obesity to lose weight UK2021. www.gov.uk/government/news/new-specialised-support-to-help-those-living-with-obesity-to-lose-weight [accessed 08/02/2021].

[7] Moyer VA U.S. Preventive Services Task Force . Screening for and management of obesity in adults: U.S. Preventive Services Task Force recommendation statement. Ann Intern Med 2012;157:373-8. 10.7326/0003-4819-157-5-201209040-00475. 22733087

[8] National Institute for Health and Care Excellence. Maintaining a Healthy Weight and Preventing Excess Weight Gain in Children and Adults. Cost Effectiveness Considerations from a Population Modelling Viewpoint. 2014, NICE. www.nice.org.uk/guidance/ng7/evidence/evidence-review-2-qualitative-evidence-review-of-the-most-acceptable-ways-to-communicate-information-about-individually-modifiable-behaviours-to-help-maintain-a-healthy-weight-or-prevent-excess-weigh-8733713.

[9] The Health Foundation. Use of primary care during the COVID-19 pandemic. 17/09/2020: The Health Foundation, 2020.

[10] Australian Bureau of Statistics. Patient Experiences in Australia: Summary of Findings, 2017-18. 2019 ed. Canberra, Australia, 2018. www.abs.gov.au/AUSSTATS/abs@.nsf/Lookup/4839.0Main+Features12017-18?OpenDocument.

[11] LeBlanc ES Patnode CD Webber EM Redmond N Rushkin M O’Connor EA . Behavioral and Pharmacotherapy Weight Loss Interventions to Prevent Obesity-Related Morbidity and Mortality in Adults: Updated Evidence Report and Systematic Review for the US Preventive Services Task Force. JAMA 2018;320:1172-91. 10.1001/jama.2018.7777. 30326501 PMC13151892

[12] Damschroder LJ Lowery JC . Evaluation of a large-scale weight management program using the consolidated framework for implementation research (CFIR). Implement Sci 2013;8:51. 10.1186/1748-5908-8-51. 23663819 PMC3656778

[13] Moher D Liberati A Tetzlaff J Altman DG PRISMA Group . Preferred reporting items for systematic reviews and meta-analyses: the PRISMA statement. PLoS Med 2009;6:e1000097. 10.1371/journal.pmed.1000097. 19621072 PMC2707599

[14] Page MJ McKenzie JE Bossuyt PM . The PRISMA 2020 statement: an updated guideline for reporting systematic reviews. BMJ 2021;372:n71. 10.1136/bmj.n71. 33782057 PMC8005924

[15] WHO. Main terminology: World Health Organization; 2004. www.euro.who.int/en/health-topics/Health-systems/primary-health-care/main-terminology [accessed 09.12.21].

[16] Abbott S Smith E Tighe B Lycett D . Group versus one-to-one multi-component lifestyle interventions for weight management: a systematic review and meta-analysis of randomised controlled trials. J Hum Nutr Diet 2021;34:485-93. 10.1111/jhn.12853. 33368624

[17] Aceves-Martins M Robertson C Cooper D REBALANCE team . A systematic review of UK-based long-term nonsurgical interventions for people with severe obesity (BMI ≥35 kg m^-2^). J Hum Nutr Diet 2020;33:351-72. 10.1111/jhn.12732. 32027072 PMC7317792

[18] Cleo G Beller E Glasziou P Isenring E Thomas R . Efficacy of habit-based weight loss interventions: a systematic review and meta-analysis. J Behav Med 2020;43:519-32. 10.1007/s10865-019-00100-w. 31529279

[19] Makin H Chisholm A Fallon V Goodwin L . Use of motivational interviewing in behavioural interventions among adults with obesity: A systematic review and meta-analysis. Clin Obes 2021;11:e12457. 10.1111/cob.12457. 33955152

[20] Patel ML Wakayama LN Bennett GG . Self-Monitoring via Digital Health in Weight Loss Interventions: A Systematic Review Among Adults with Overweight or Obesity. Obesity (Silver Spring) 2021;29:478-99. 10.1002/oby.23088. 33624440 PMC12838191

[21] Kettle VE Madigan CD Coombe A . Effectiveness of physical activity interventions delivered or prompted by health professionals in primary care settings: systematic review and meta-analysis of randomised controlled trials. BMJ 2022;376:e068465. 10.1136/bmj-2021-068465. 35197242 PMC8864760

[22] Covidence [program]. Melbourne, 2021.

[23] Welzel FD Bär J Stein J . Using a brief web-based 5A intervention to improve weight management in primary care: results of a cluster-randomized controlled trial. BMC Fam Pract 2021;22:61. 10.1186/s12875-021-01404-0. 33794781 PMC8017625

[24] Carrington MJ Zimmet PZ . Nurse co-ordinated health and lifestyle modification for reducing multiple cardio-metabolic risk factors in regional adults: outcomes from the MODERN randomized controlled trial. Eur J Cardiovasc Nurs 2022; 21:26-35. 10.1093/eurjcn/zvab042. 33899090

[25] Fernández-Ruiz VE Ramos-Morcillo AJ Solé-Agustí M Paniagua-Urbano JA Armero-Barranco D . Effectiveness of an Interdisciplinary Program Performed on Obese People Regarding Nutritional Habits and Metabolic Comorbidity: A Randomized Controlled Clinical Trial. Int J Environ Res Public Health 2020;17:E336. 10.3390/ijerph17010336. 31947784 PMC6981546

[26] Bräutigam-Ewe M Lydell M Bergh H Hildingh C Baigi A Månsson J . Two-year weight, risk and health factor outcomes of a weight-reduction intervention programme: Primary prevention for overweight in a multicentre primary healthcare setting. Scand J Prim Health Care 2020;38:192-200. 10.1080/02813432.2020.1753379. 32362238 PMC8570757

[27] Little P Stuart B Hobbs FR . An internet-based intervention with brief nurse support to manage obesity in primary care (POWeR+): a pragmatic, parallel-group, randomised controlled trial. Lancet Diabetes Endocrinol 2016;4:821-8. 10.1016/S2213-8587(16)30099-7 27474214

[28] Yardley L Ware LJ Smith ER . Randomised controlled feasibility trial of a web-based weight management intervention with nurse support for obese patients in primary care. Int J Behav Nutr Phys Act 2014;11:67. 10.1186/1479-5868-11-67. 24886516 PMC4045942

[29] Christian JG Bessesen DH Byers TE Christian KK Goldstein MG Bock BC . Clinic-based support to help overweight patients with type 2 diabetes increase physical activity and lose weight. Arch Intern Med 2008;168:141-6. 10.1001/archinternmed.2007.13. 18227359

[30] Christian JG Byers TE Christian KK . A computer support program that helps clinicians provide patients with metabolic syndrome tailored counseling to promote weight loss. J Am Diet Assoc 2011;111:75-83. 10.1016/j.jada.2010.10.006. 21185968

[31] Martin PD Dutton GR Rhode PC Horswell RL Ryan DH Brantley PJ . Weight loss maintenance following a primary care intervention for low-income minority women. Obesity (Silver Spring) 2008;16:2462-7. 10.1038/oby.2008.399. 18787526 PMC2753427

[32] Tsai AG Wadden TA Rogers MA Day SC Moore RH Islam BJ . A primary care intervention for weight loss: results of a randomized controlled pilot study. Obesity (Silver Spring) 2010;18:1614-8. 10.1038/oby.2009.457. 20019680

[33] Wadden TA Walsh OA Berkowitz RI . Intensive Behavioral Therapy for Obesity Combined with Liraglutide 3.0 mg: A Randomized Controlled Trial. Obesity (Silver Spring) 2019;27:75-86. 10.1002/oby.22359. 30421856 PMC6800068

[34] Kumanyika SK Morales KH Allison KC . Two-Year Results of Think Health! ¡Vive Saludable!: A Primary Care Weight-Management Trial. Obesity (Silver Spring) 2018;26:1412-21. 10.1002/oby.22258. 30160061 PMC6143399

[35] Appel LJ Clark JM Yeh H-C . Comparative effectiveness of weight-loss interventions in clinical practice. N Engl J Med 2011;365:1959-68. 10.1056/NEJMoa1108660. 22085317 PMC4074540

[36] Baer HJ Rozenblum R De La Cruz BA . Effect of an Online Weight Management Program Integrated With Population Health Management on Weight Change: A Randomized Clinical Trial. JAMA 2020;324:1737-46. 10.1001/jama.2020.18977. 33141209 PMC7610192

[37] Katzmarzyk PT Martin CK Newton RL Jr . Weight Loss in Underserved Patients - A Cluster-Randomized Trial. N Engl J Med 2020;383:909-18. 10.1056/NEJMoa2007448. 32877581 PMC7493523

[38] Nanchahal K Power T Holdsworth E . A pragmatic randomised controlled trial in primary care of the Camden Weight Loss (CAMWEL) programme. BMJ Open 2012;2:e000793. 10.1136/bmjopen-2011-000793 22561352 PMC3353130

[39] Ross R Lam M Blair SN . Trial of prevention and reduction of obesity through active living in clinical settings: a randomized controlled trial. Arch Intern Med 2012;172:414-24. 10.1001/archinternmed.2011.1972. 22371872

[40] RevMan [program]. 5.4 version: Copenhagen, 2014.

[41] Jolly K Lewis A Beach J . Comparison of range of commercial or primary care led weight reduction programmes with minimal intervention control for weight loss in obesity: lighten Up randomised controlled trial. BMJ 2011;343:d6500. 10.1136/bmj.d6500. 22053315 PMC3208022

[42] Jebb SA Ahern AL Olson AD . Primary care referral to a commercial provider for weight loss treatment versus standard care: a randomised controlled trial. Lancet 2011;378:1485-92. 10.1016/S0140-6736(11)61344-5 21906798 PMC3207352

[43] Sterne JAC Savović J Page MJ . RoB 2: a revised tool for assessing risk of bias in randomised trials. BMJ 2019;366:l4898. 10.1136/bmj.l4898. 31462531

[44] Gomez-Huelgas R Jansen-Chaparro S Baca-Osorio AJ Mancera-Romero J Tinahones FJ Bernal-López MR . Effects of a long-term lifestyle intervention program with Mediterranean diet and exercise for the management of patients with metabolic syndrome in a primary care setting. Eur J Intern Med 2015;26:317-23. 10.1016/j.ejim.2015.04.007. 25907985

[45] Delahanty LM Chang Y Levy DE . Design and participant characteristics of a primary care adaptation of the Look AHEAD Lifestyle Intervention for weight loss in type 2 diabetes: The REAL HEALTH-diabetes study. Contemp Clin Trials 2018;71:9-17. 10.1016/j.cct.2018.05.018. 29803816 PMC6067988

[46] Tárraga Marcos ML Panisello Royo JM Carbayo Herencia JA . [Analysis of clinical relevance applied to 3methods of reducing weight in overweight or obesity followed-up for one year]. Hipertens Riesgo Vasc 2018;35:5-14. 10.1016/j.hipert.2017.06.004. 28916164

[47] Beeken RJ Leurent B Vickerstaff V . A brief intervention for weight control based on habit-formation theory delivered through primary care: results from a randomised controlled trial. Int J Obes (Lond) 2017;41:246-54. 10.1038/ijo.2016.206. 27867204 PMC5300101

[48] Phelan S Hagobian T Brannen A . Effect of an Internet-Based Program on Weight Loss for Low-Income Postpartum Women: A Randomized Clinical Trial. JAMA 2017;317:2381-91. 10.1001/jama.2017.7119. 28632867 PMC5815021

[49] Rodriguez-Cristobal JJ Alonso-Villaverde C Panisello JM . Effectiveness of a motivational intervention on overweight/obese patients in the primary healthcare: a cluster randomized trial. BMC Fam Pract 2017;18:74. 10.1186/s12875-017-0644-y. 28633627 PMC5477747

[50] Cai R Chao J Li D Zhang M Kong L Wang Y . Effect of community-based lifestyle interventions on weight loss and cardiometabolic risk factors in obese elderly in China: A randomized controlled trial. Exp Gerontol 2019;128:110749. 10.1016/j.exger.2019.110749. 31644921

[51] Conroy MB Sward KL Spadaro KC . Effectiveness of a physical activity and weight loss intervention for middle-aged women: healthy bodies, healthy hearts randomized trial. J Gen Intern Med 2015;30:207-13. 10.1007/s11606-014-3077-5. 25391601 PMC4314485

[52] Kanke S Kawai T Takasawa N Mashiyama Y Ishii A Kassai R . Interventions for body weight reduction in obese patients during short consultations: an open-label randomized controlled trial in the Japanese primary care setting. Asia Pac Fam Med 2015;14:5. 10.1186/s12930-015-0022-7. 26015773 PMC4443656

[53] Pritchard DA Hyndman J Taba F . Nutritional counselling in general practice: a cost effective analysis. J Epidemiol Community Health 1999;53:311-6. 10.1136/jech.53.5.311. 10396539 PMC1756872

[54] Logue E Sutton K Jarjoura D Smucker W Baughman K Capers C . Transtheoretical model-chronic disease care for obesity in primary care: a randomized trial. Obes Res 2005;13:917-27. 10.1038/oby.2005.106. 15919846

[55] Bennett GG Steinberg D Askew S . Effectiveness of an App and Provider Counseling for Obesity Treatment in Primary Care. Am J Prev Med 2018;55:777-86. 10.1016/j.amepre.2018.07.005. 30361140 PMC6388618

[56] Taheri S Zaghloul H Chagoury O . Effect of intensive lifestyle intervention on bodyweight and glycaemia in early type 2 diabetes (DIADEM-I): an open-label, parallel-group, randomised controlled trial. Lancet Diabetes Endocrinol 2020;8:477-89. 10.1016/S2213-8587(20)30117-0. 32445735

[57] Lean ME Leslie WS Barnes AC . Primary care-led weight management for remission of type 2 diabetes (DiRECT): an open-label, cluster-randomised trial. Lancet 2018;391:541-51. 10.1016/S0140-6736(17)33102-1. 29221645

[58] Moore H Summerbell CD Greenwood DC . Improving management of obesity in primary care: cluster randomised trial. BMJ 2003;327:1085. 10.1136/bmj.327.7423.1085. 14604931 PMC261745

[59] Huseinovic E Bertz F Leu Agelii M Hellebö Johansson E Winkvist A Brekke HK . Effectiveness of a weight loss intervention in postpartum women: results from a randomized controlled trial in primary health care. Am J Clin Nutr 2016;104:362-70. 10.3945/ajcn.116.135673. 27413127

[60] Wing RR Lang W Wadden TA Look AHEAD Research Group . Benefits of modest weight loss in improving cardiovascular risk factors in overweight and obese individuals with type 2 diabetes. Diabetes Care 2011;34:1481-6. 10.2337/dc10-2415. 21593294 PMC3120182

[61] LeBlanc EL Patnode CD Webber EM . U.S. Preventive Services Task Force Evidence Syntheses, formerly Systematic Evidence Reviews. Behavioral and Pharmacotherapy Weight Loss Interventions to Prevent Obesity-Related Morbidity and Mortality in Adults: An Updated Systematic Review for the US Preventive Services Task Force. Rockville (MD). Agency for Healthcare Research and Quality, 2018. 30354042

[62] Ahern AL Wheeler GM Aveyard P . Extended and standard duration weight-loss programme referrals for adults in primary care (WRAP): a randomised controlled trial. Lancet 2017;389:2214-25. 10.1016/S0140-6736(17)30647-5. 28478041 PMC5459752

[63] de Koning L Chiuve SE Fung TT Willett WC Rimm EB Hu FB . Diet-quality scores and the risk of type 2 diabetes in men. Diabetes Care 2011;34:1150-6. 10.2337/dc10-2352. 21464460 PMC3114491

[64] World Health Organization. Obesity and Overweight, 2021, www.who.int/news-room/fact-sheets/detail/obesity-and-overweight

[65] Starfield B Shi L Macinko J . Contribution of primary care to health systems and health. Milbank Q 2005;83:457-502. 10.1111/j.1468-0009.2005.00409.x. 16202000 PMC2690145

[66] Sturgiss E Madigan CD Klein D Elmitt N Douglas K . Metabolic syndrome and weight management programs in primary care: a comparison of three international healthcare systems. Aust J Prim Health 2018;24:372-7. 10.1071/PY18021. 30056826

[67] Dewhurst A Peters S Devereux-Fitzgerald A Hart J . Physicians’ views and experiences of discussing weight management within routine clinical consultations: A thematic synthesis. Patient Educ Couns 2017;100:897-908. 10.1016/j.pec.2016.12.017. 28089308

[68] Sturgiss E Haesler E Elmitt N van Weel C Douglas K . Increasing general practitioners’ confidence and self-efficacy in managing obesity: a mixed methods study. BMJ Open 2017;7:e014314. 10.1136/bmjopen-2016-014314. 28132016 PMC5278274

[69] Gafoor R Booth HP Gulliford MC . Antidepressant utilisation and incidence of weight gain during 10 years’ follow-up: population based cohort study. BMJ 2018;361:k1951. 10.1136/bmj.k1951. 29793997 PMC5964332

[70] Little P Stuart B Hobbs FR . Randomised controlled trial and economic analysis of an internet-based weight management programme: POWeR+ (Positive Online Weight Reduction). Health Technol Assess 2017;21:1-62. 10.3310/hta21040 28122658 PMC5292642

[71] Kumanyika SK Fassbender JE Sarwer DB . One-year results of the Think Health! study of weight management in primary care practices. Obesity (Silver Spring) 2012;20:1249-57. 10.1038/oby.2011.329. 22051940

[72] Huseinovic E Bertz F Brekke HK Winkvist A . Two-year follow-up of a postpartum weight loss intervention: Results from a randomized controlled trial. Matern Child Nutr 2018;14:e12539. 10.1111/mcn.12539. 28984033 PMC6865986

