# Supplementary material for: Effectiveness of weight management interventions for adults delivered in primary care: systematic review and meta-analysis of randomised controlled trials
Source: BMJ. 2022 May 31;377:e069719. doi: 10.1136/bmj-2021-069719 (PMC9150078; doi:10.1136/bmj-2021-069719)
Supplement: Supplementary file 1 — Supplementary information: Additional information, tables, and figures [file madc069719.ww.pdf]

Supplementary files

Supplementary File 1: Medline search 17th August 2021

1 Obesity/ 192464
2 Obesity, Morbid/ 22525
3 Overweight/ 27513
4 Obesity, Metabolically Benign/ 282
5 Weight loss/ 38989
6 obes$.m_titl. 132663
7 overweight.m_titl. 18218
8 weight.m_titl. 108318
9 (adipos$ or body fat).m_titl. 46877
10 (obes$ or overweight or weight loss).ti,ab. 406126
11 limit 10 to (“in data review” or in process or “pubmed not medline”) 50034
12 1 or 2 or 3 or 4 or 5 or 6 or 7 or 8 or 9 or 11 412754
13 Weight Reduction Programs/ 2515
14 Behavior Therapy/ 28859
15 Cognitive Therapy/ 27380
16 Counseling/ 37398
17 Directive Counseling/ 2408
18 Self-Help Groups/ 9335
19 counsel$.ti,ab. 117522
20 (1ehave$ adj3 (therap$ or program$ or intervention$)).ti,ab. 54632
21 Health Education/ 62116
22 Diet, Reducing/ 11289
23 Diet, Fat-Restricted/ 3814
24 Caloric Restriction/ 6573
25 (diet$ adj counsel$).ti,ab. 1912
26 (diet$ adj education$).ti,ab. 695
27 (nutrition$ adj counsel$).ti,ab. 2130
28 (nutrition$ adj education$).ti,ab. 5861
29 Diet Therapy/ 10783
30 (nutrition$ adj intervention$).ti,ab. 7803
31 (diet$ adj (modif$ or therapy or intervention$ or strateg$)).ti,ab. 18326
32 ((diet or dieting or slim$) adj (club$ or organi?ation$)).ti,ab. 32
33 (weight reduc$ adj diet$).ti,ab. 294
34 Exercise/ 121811
35 Exercise Therapy/ 43992
36 Motor Activity/ 98455
37 Physical Conditioning, Human/ 2730
38 Physical Fitness/ 28326
39 physical activity.ti,ab. 120016
40 (exercise adj3 (therap$ or program$ or intervention$)).ti,ab.33457
41 ((lifestyle or lifestyle) adj (modification$ or intervention$)).ti,ab. 14801
42 13 or 14 or 15 or 16 or 17 or 18 or 19 or 20 or 21 or 22 or 23 or 24 or 25 or 26 or 27 or 28 or 29 or 30 or 31 or 32 or 33 or 34 or 35 or 36 or 37 or 38 or 39 or 40 or 41 669360
43 12 and 42 61987
44 Obesity/dh, th, dt, rh [Diet Therapy, Therapy, Rehabilitation] 33977

45 Obesity, Morbid/dh, th, dt, rh 1720
46 Overweight/dh, th, dt, rh 4345
47 (weight loss adj (intervention$ or program$ or trial$)).ti,ab. 4541
48 (weight reduc$ adj (intervention$ or program$ or trial$)).ti,ab. 821
49 (weight management adj (intervention$ or program$ or trial$)).ti,ab. 1875
50 (weight control adj (intervention$ or program$ or trial$)).ti,ab. 550
51 (weight loss maintenance adj (intervention$ or program$ or trial$)).ti,ab. 114
52 43 or 44 or 45 or 46 or 47 or 48 or 49 or 50 or 51 85095
53 limit 52 to "all child (0 to 18 years)" 21000
54 limit 52 to "all adult (19 plus years)" 37043
55 53 not 54 12117
56 52 not 55 72978
57 limit 56 to animals 10380
58 limit 56 to humans 59178
59 57 not 58 5670
60 56 not 59 67308
61 clinical trials as topic/ or controlled clinical trials as topic/ or randomized controlled trials as topic/ or meta-analysis as topic/ 357980
62 (clinical trial or controlled clinical trial or meta analysis or randomized controlled trial).pt. 1017190
63 Random$.ti,ab. 1244756
64 control groups/ or double-blind method/ or single-blind method/ 197825
65 clinical trial$.ti,ab. 407889
66 controlled trial$.ti,ab. 260595
67 controlled trial$.ti,ab. 260595
68 61 or 62 or 63 or 64 or 65 or 66 or 672154858
69 60 and 68 18404
70 limit 69 to yr="2018 -Current" 4501

Supplementary File 2: Planned analysis of subgroups and results

Planned analysis

A further aim was to examine weight change at programme end; but as most studies lasted a minimum of 12 months this analysis would be unlikely to add to the interpretation. There was a number of pre-specified outcomes. Firstly, we considered examining anxiety, depression, and quality of life but only three trials had measures of these outcomes[29-31]. We pre-specified that we aimed to explore dropout rates but data were reported differently by trials. Some trials reported only those that did not have weight data at follow-up, and we could not distinguish between those who had dropped out because they did not like the intervention and those that were not followed-up. Instead, we compared those at high risk of bias for ≥20 % attrition with those with low attrition.

Results

Weight change by country

There was no difference (p=0.78) in country but the confidence intervals of trials conducted in the UK overlapped suggesting that there may be no difference between comparator and intervention groups in the UK (mean difference -1.4 kg, 95% CI -3.1 to 0.3, I^2 85%, Tau^2 4.22 p=0.10) whereas for the US and Spain, the confidence intervals suggest most participants lost weight (Supplementary Figure 5).

Weight change by intervention delivery practitioner

There were significant differences in the subgroups (p=0.005) to explore the effects of who delivered the intervention. Interventions delivered by non-medical practitioners (e.g., non-medical assistants or health coaches) had a mean difference of -2.0 kg (95% CI -2.9 to -1.1, I^2 69%, Tau^2 1.3, n=8 trials, n=3,039). Interventions delivered by GPs had a mean difference of

-1.1 kg (95% CI -1.9 to -0.3 *I^2 0%,* Tau^2 *0,* n= 4 trials, n=788) and nurse delivered interventions had a mean difference of -0.5 kg (95% CI -1.2 to 0.3, *I^2*5%, Tau^2 *0.03* n=4 trials, n=1,004). The 'other' practitioner's group had a mean difference of -2.3 kg (95% CI -3.2 to -1.4, *I^2* 86%, Tau^2 *2.78,* n=14 trials, n= 4,107) (Supplementary Figure 2).

Weight change by number of contacts

Participants who received more contacts (12 or more) within interventions lost significantly more weight (-2.4 kg, 95% CI -3.0 to -1.7, *I^2* 82%, Tau^2 2.11 p<0.001, n= 20) than those who had less contacts (-0.7 kg, 95% CI -1.2 to -0.1 *I^2* 25%, Tau^2 *0.22* n=10) (Supplementary Figure 6). Using a univariable meta-regression to explore the effect further, there was evidence that an increase in number of sessions was associated with a decrease in weight (-0.07 (95% CI: -0.12 to -0.02, P=0.01). The association remained after adjustment for who delivered the intervention (-0.06 (95% CI: -0.12 to 0.00, P=0.046).

Weight change by attrition

There were five trials[28 33 40 47 53] with attrition ≥20 % that had the potential to bias the results and the mean difference in weight at last follow-up was -0.8 kg (95% CI -1.3 to -0.2) compared to the other trials with <20% attrition of -2.1 kg (95% CI -2.8 to -1.5).

Supplementary Table 1: Characteristics of included trials

Study, year, country, design	N randomised	Inclusion Criteria	Main Exclusion Criteria	Percentage female	Mean age (SD)	Mean BMI (SD)	Intervention	Intervention delivered by	Training for staff	Comparator	Intervention duration and follow, ups
Appel, 2011, US, RCT [1]	415	Aged >21 years, one or more cardiovascular risk factors and with obesity.	Lost at least 5% of weight or taking medications that affect weight.	63.6	54 (10.2)	36.6 (5.0)	Web based self-monitoring and feedback and in person or remote counselling (two intervention groups). PCP's provided supportive role and reviewed progress at each routine consultation. 17 contacts for in person and 20+ remote contacts.	PCP, coaches employed by university (face to face support) and coaches employed by private company (remote support).	Training provided to health coaches in MI and behaviour change. PCP received training about the reinforcement of behavioural changes in routine visits.	Usual Care	24 months<br><br>F-up: 6,12,24 months
Baer, 2020, US, Cluster RCT[2]	624	Aged 20 to 70 years, BMI 27 to 40 kg/m^2, and a diagnosis of hypertension or type 2 diabetes.	Bariatric surgery, lost at least 5% of weight or taking medications that affect weight.	60.3	59.4 (8.5)	32.6 (3.3)	Online intervention and support from a population health manager and a brief session with a dietician. Monthly phone calls with the manager who uploaded reports every month. 14 + contacts.	Population health manager (non-clinical member of staff), dietician and online programme.	NR	Usual care	12 months<br><br>F-up 12 months

Beeken, 2017, UK, RCT[3]	537	Aged ⩾18 years, BMI ⩾30 kg/m²	Active psychotic illness, pregnant or terminally ill.	65.7	median 59.4 IQR- 48.7–66.8	median 35 IQR 32.6 to 39.2	10 top tips leaflet, logbook for self-monitoring of target behaviours and weight. A single 30 min session was allocated to take patients through the leaflet using a flip chart and defined script. 1 contact.	Nurses or healthcare assistants	Attended a training session, and were provided with a script to enable them to deliver the intervention in a standardised way.	Usual Care	1 single appointment<br><br>F-up: 3, 6, 12,18 and 24 months
Bennett, 2018, US, RCT[4]	351	Aged 21 to 65 years, BMI of 30.0–44.9 kg/m² and a weight ≤ 330 lb, diagnosed hypertension, type 2 diabetes, and/or hyperlipidaemia	Pregnancy, being ≤ 12 months postpartum, or condition that would affect weight.	68.1	50.7 (8.9)	35.9 (3.9)	Behavioural intervention via an app with 18 weight loss support calls with a dietician and 6 brief weight loss consultations with PCP. 18+ contacts.	Dietician, PCP and app	Dieticians received: 2-day training session and biannual refresher trainings. PCPS annual in-service trainings on weight loss counselling.	Usual Care	12 months
Bräutigam-Ewe, 2020, Sweden, RCT[5]	286	Aged 40 to 65 years, BMI of 28–35 kg/m²	Undergoing treatment that could be affected by participating, known drug addictions.	80.8	55.7 (7.1)	31.4 (NR)	Motivational interviewing, a grocery store lecture, website communication and weekly e-mails. The participants participated in MI conversations three times about lifestyle habits. 7+ contacts.	Nurse	Two days of training in MI	Usual care	6 months<br><br>F-up: 24 months
Cai, 2019, China, RCT [6]	480	Aged >60 years and BMI > 28 kg/m²	Cognitive defects, severe psychological disorders or mental illnesses, cancer, recent CVD and other severe chronic diseases.	54	67 (5.0)	30.1 (1.8)	Group and individual sessions. Behavioural intervention, classroom-style sessions for 2 h every two weeks in the first 12 months and every month from month 13 to 24 with ongoing telephone support, and health promoting materials. 36+ contacts.	PCP, dieticians, over the phone and in person.	NR	Usual care	2 years<br><br>F-up: 18 and 24 months

Carrington, 2021, Australia, RCT[7]	276	Aged 40 to 70 years with any three or more risk factors for metabolic syndrome	Chronic disease that would affect participation.	61	57.5 (7.5)	31.7 (5.5)	Face to face and telephone counselling. The number of visits depended on risk stratification and need. Maximum 8 visits in 2 years. 2-8 contacts.	Nurses	NR	Usual care	24-month intervention F-up: 12 and 24 months
Christian, 2008, US, RCT[8]	310	Aged 18-75 years with Type 2 diabetes. BMI ⩾25 kg/m^2, uninsured, Medicaid eligible, or Medicare beneficiaries	Substance use or abuse, severe arthritis or other medical conditions limiting physical activity, recent CVD, bariatric surgery.	66	53.4 (11)	35.1 (6.9)	Computer based self-management programme with PCP feedback. 4 contacts.	Online and physician visits	3-hour training session to provide brief motivational interviewing counselling to help patients make changes.	Usual care and leaflets	9 months F-up: 6 and 12 months
Christian, 2011, US, Cluster RCT[9]	279	Aged 18 to 75 years; waist circumference at 35 inches for women or 40 inches for men, or BMI> 25 kg/m^2 and two or more features of the metabolic syndrome	Substance use/abuse; severe arthritis or other medical conditions limiting physical activity; recent CVD.	68.4	49.6 (12.4)	34.3 (7.4)	Computer based self-management programme with PCP feedback. 1 + usual care visits.	Online and physician visits	3-hour training session to provide brief motivational interviewing counselling to help patients make changes.	Usual care	12 months F-up: 12 months

Conroy, 2014, US, RCT[10]	99	Women aged 45–65 years, BMI ≥ 25 kg/m^{2}, physically inactive	Unstable cardiac or pulmonary disease, poorly controlled hypertension, unable to perform moderate PA.	100	53.9 (5.4)	34.7 (5.9)	12 weekly group sessions of behaviour change. 12 contacts.	Face to face by physician at practice and two others not affiliated to practice.	NR	Self-guided manual and pedometer	12 weeks<br><br>F-up: 12 months
Delahanty, 2020, US, RCT [11]	211	Adults with type 2 diabetes, BMI > 25 kg/m^{2}. HbA1c 6.5 to < 11.5%, systolic/diastolic blood pressure < 160/100 mmHg.	Pregnancy, participation in a weight loss program, weight change of > 3% in the previous month, bariatric surgery, medications that affect weight or psychiatric conditions.	55.5	61.2 (10.1)	35.1 (5.5)	Behavioural intervention based on DPP but adapted for primary care group and individual sessions. One intervention group delivered face to face the other over the phone. 25-28 contacts.	Dietician	NR	Usual care	2 years<br><br>F-up: 6 and 12 months
Fernández-Ruiz, 2016, Spain, RCT [12 13]	74	Adults, BMI > 25 kg/m^{2}	Comorbidities with other pathologies (depression, cancer, fibromyalgia and others which could interfere with intervention.	50	61.1 (9.0)	33.4 (4.2)	4 weekly sessions of physical activity lasting 40 minutes. Psychologists conducted a monthly 60-minute cognitive behavioural therapy session. 40 contacts.	Interdisciplinary team- led by the practice nurse with psychologist, physician, physical activity monitor and nutritionist.	Health education- 60-minute monthly session focusing on treating obesity and its comorbidities.	Usual care	12 months<br><br>F-up: 6 12 and 24 months
Gomez-Hueglas, 2015,	601	Aged 18-80 years, with metabolic syndrome	NR	44.9	53.8 (14.3)	31.1 (4.7)	Mediterranean diet, extensive PA. 9 medical visits and 18 nursing visits.	Nurse, PCP	NR	Usual care	3 years<br>F-up: 3 years

Spain, RCT [14]							Group and individual sessions. 27 contacts				
Huseinovic, 2016, Sweden, RCT[15 16]	110	BMI >27 kg/m^2 at 6–15 wk postpartum, women.	Serious disease in woman or child, participation in another weight trial.	100	32.2 (4.6)	31.7 (3.7)	BWMP focused on postpartum, one session and then telephone contacts bi-weekly. Then standardised monthly emails up to 12 months. 7 contacts.	Dietician	NR	Leaflet on healthy eating	12 months<br><br>F-up: 12 months and 2 years
Jolly, 2011, UK, RCT [17 18]	170	Aged >18 years old, BMI> 30 kg/m^2	Pregnant, no medical contraindications for programmes.	27.1	50 (13.8)	NR	One to one sessions focused on behaviour change. 12 contacts.	Nurse or Dietician (as available locally)	3-day training course on weight management in adults delivered by dietitians experienced in the management of obesity.	Vouchers for 12 free sessions at a leisure centre	12 weeks<br><br>F-up: 12 weeks, 12 months
Kanke, Japan, 2015, RCT[19]	50	Aged 30–69 years, BMI ≥25 kg/m^2 who visited GP for hypertension, dyslipidemia, and/or type 2 diabetes mellitus	A history of cancer or psychological disease, or those prescribed hormone therapy.	36	54.8 (6.7)	28.1 (2.1)	Individual counselling by PCP at regular consultations. 1+ contacts.	GP	NR	Brief advice about ideal body weight	1 year

Katzmarzyk, 2020, US, Cluster RCT [20 21]	803	Aged 20-75 years old, BMI 30-50 kg/m²	Participating in a weight loss program, weight loss medication, bariatric surgery within the last 2 years, or had lost > 10 lb of weight within the last 6 months.	84.4	49.4 (13.1)	37.2 (4.7)	High-intensity lifestyle intervention program based on DPP, Look AHEAD and CALERIE. Consisted of weekly sessions (16 face-to-face sessions and 6 via phone) during the first 6 months and at least monthly sessions for the remaining 18 months. 40 contacts.	Trained health coaches	Received further training in the management of obesity and related comorbidities, fundamentals of health literacy and patient communication and education.	Usual care	24 months<br><br>F-up: 6,12,18,24 months
Kumanyika, 2012 and 2018, US, RCT[22 23]	261	Aged 18–70 years, with a BMI ≥27 kg/m² and ≤55 kg/	Pregnant or lactating; medications that affect weight, undergoing active cancer treatment; and having unstable CVD or significant mental health conditions.	84.3	47.2 (NR)	37.2 (6.4)	DPP individual based counselling, 4 sessions with PCP and 12 individual coaching sessions. 17 contacts.	PCP and auxiliary staff acting as coaches	Training in behavioural counselling for PCP and auxiliary staff by research staff.	Usual care	1 year F-up: 1 year
Lean, 2018 UK, Cluster RCT[24 25]	299	Aged 20–65 years, had been diagnosed with type 2 diabetes within the previous 6 years, BMI of 27–45 kg/m²	Insulin use, a glycated haemoglobin (HbA1c) concentration of 12%, weight loss of more than 5 kg within the past 6 months, substance abuse, known cancer, Recent	41	54.4 (7.6)	34.6 (4.4)	Behavioural weight management programme and TDR for 3 months and reintroduction of food 2-8 weeks. Monthly behavioural support. 35 contacts.	Nurse or Dietician (as available locally)	8 hours structured training by research dieticians	Usual Care	2 years<br><br>F-up: 12 and 24 months

			CVD, obesity medications.								
Little, 2017, UK, RCT,[26 27]	826	Aged >18 years or older, BMI >30 kg/m² (or ≥28 kg/m² with hypertension, hypercholesterolaemia, or diabetes)	Severe mental health problems, illness, pregnant or breastfeeding, perceived inability to walk 100 m.	63.6	53.7 (13.1)	36.7 (5.7)	24 web-based sessions over 6 months and email reminders. Two intervention groups Power F: 3 scheduled (and four optional) face-to-face nurse support sessions POWER R: 3 phone or email contacts and up to 2 optional phone/ email contacts in the first 6 months. 3-7 contacts.	Nurse and online	NR	Two printable web-based pages with brief structured advice	6 months<br><br>F-up: 6 and 12 months
Logue, 2005, US, RCT[28]	665	Aged 40 to 69 years BMI > 27 kg/m^2 or elevated waist-to-hip ratios.	Pregnancy, lactation, 6 months postpartum, or use of a wheelchair for mobility. Severe heart or lung disease.	68.9	NR	NR	Individual, telephone counselling plus personalised mailings. 4 x individual sessions and 24 phone calls. 24 + contacts.	PCP, dietitian and weight loss advisors.	NR	Usual care	2 years<br><br>F-up: 6,12,18 ,24 months
Martin, 2008, US, Cluster RCT[29]	144	Aged 18 to 65 years, BMI ≥ 25 kg/m^2, classified as low income.	Free of serious or uncontrolled medical conditions.	100	41.8 (12.0)	38.9 (7.6)	6 PCP visits, 5 intense and then one at 6 months, 6 contacts.	PCP	2 hours of instruction on general obesity treatment, the physicians providing tailored interventions received an additional 5 h of training, which addressed the assessment of stage of change, motivational interviewing, and	Usual care	6 months<br><br>F-up: 6,9,12, 18 months

									techniques for the behavioural treatment of obesity.		
Moore, 2003, UK, Cluster RCT[30]	843	Aged 16 to 64 years BMI ≥ 30 kg/m^2	NR	50	48.6 (11.6)	36.9 (5.7)	Training programme for PCP staff about weight management, see patients every 2 weeks until lost 10% of body weight. 1 + contacts.	PCP or nurse	Three 90 minute sessions.	Usual care	6 months<br><br>F-up: 3,12,18 months
Nanchahal, 2012, UK, RCT[31]	381	BMI >25 kg/m^2 and adults	Pregnancy, lactation, diagnosis of renal failure, use of pacemaker, recent diagnosis of cancer.	72.2	48.8 (14.8)	33.5 (5.5)	Behavioural weight management intervention, 14 sessions for 30 minutes over 36 weeks. 14 contacts.	Health trainers at practice	2 days of training and further meetings every 3-4 months.	Usual care	36 weeks<br><br>F-up: 6,12 months
Phelan, 2017, US, Cluster RCT[32]	371	Post-partum women, aged 18-40 years with low income, exceed pre-pregnancy weight by at least 6.8 kg and BMI>22 kg/m^2	NR	100	28.1 (5.4)	31.7 (5.1)	During usual consultations promotion of programme and then monthly group meetings and access to online BWMP. 12 contacts.	Dieticians and public health aides, online and monthly sessions by researchers.	NR	Usual care	12 months
Pritchard, 1999, Australia, RCT[33]	182	Aged 25 to 65 years, pre-existing diagnosis of overweight, hypertension or type 2 diabetes.	Mentally ill, intellectually handicapped, terminally ill, acutely ill, pregnant or participating in other health	72.5	NR	NR	3 GP consultations to encourage weight loss. 6 sessions with study dietician. 9 contacts.	Study dietician and GP	NR	Given results of weight	12 months

			education programmes.								
Rodriguez-Cristobal, 2017, Spain, Cluster RCT[34]	864	Aged 30 to 70 years, BMI>25 kg/m^2	Severe clinical pathology, secondary obesity, severe sensorial disorders, or psychiatric disorders.	77.2	56.5 (16.5)	34.1 (4.8)	Group motivation sessions by nurse, 32 contacts.	Practice Nurse	Training from expert psychologists, consisting of a basic training strategy, and focusing on group motivation for life-style changes.	Usual care	24-month intervention<br><br>F-up: 12 and 24 months
Ross, 2012, Canada, RCT[35]	490	Aged 25–75 years, BMI 25–39.9 kg/m2 and abdominally obese (waist circumference >102 or >88 cm in men and women	Significant CVD, insulin-dependent diabetes mellitus, pregnancy or physical impairment.	70	51.8 (11.4)	32.3 (4.2)	Behavioural weight management intervention, 21 sessions over 12 months. 33 contacts.	Health advisor	NR	Usual care	12-months<br><br>F-up: 6.12.18 .24 months
Taheri, 2020, Qatar, RCT[36]	158	Aged 18-50 years, reported a diagnosis of type 2 diabetes within the previous 3 years had a BMI of > 27 kg/m².	Recent CVD, chronic kidney disease, pregnant, lactating, severe psychiatric disorder, uncontrolled depression, epilepsy.	25.3	42.1 (5.6)	34.9 (5.5)	Intensive lifestyle intervention. 12-week total diet replacement phase, followed by a 12-week structured food reintroduction phase. After this, participants managed their own energy restricted food intake and lifestyle changes for 6 months. 18 contacts.	Trained dieticians, personal trainers and physicians.	None	Usual medical diabetes care according to clinical guidelines.	12-months

TarragaMarcos, 2018, Spain, RCT[37]	180	Aged 30 to 70 years, BMI > 25 kg/m^2	Severe diseases, secondary obesity, diseases with severe sensory impairments or severe psychiatric illnesses.	56.6	49.9 (6.7)	30.6 (3.4)	G1: Motivational behavioural change intervention with nurse using online platform, once every two weeks and then monthly from weeks 12-32. 29 contacts<br><br>G2: 5 visits over a year to review data uploaded to online platform. 5 contacts.	Nurse and online programme	NR	Usual care	12 months<br><br>F-up:1 year
Tsai, 2009, US, RCT[38]	50	BMI 27–50 kg/m^2	Medical conditions that contraindicated weight loss, medications affect weight, substance abuse, or serious psychiatric illness.	NR	51.9 (12.2)	36.5 (SE 1.1)	Adapted DPP, eight brief (15–20 min) individual visits with a Medical Assistant at weeks 0, 2, 4, 8, 12, 16, 20, and 24. 8 contacts.	Medical Assistants and PCP	Training (3 hours) general education about obesity and role plays, using the DPP handouts, which were conducted until MAs demonstrated mastery of the materials. PCPs provided an overview of the handouts that they distributed at quarterly visits.	Usual Care	12 months<br><br>F-up: 6 and 12 months
Wadden, 2011, US, RCT[39]	261	Aged> 21 years, BMI 30 to 50 kg/m^2, and at least two of five components of the metabolic syndrome	Recent CVD, other medical conditions contraindicating weight loss, medications that affect body weight, psychotic illness, substance abuse.	79.6	51.5 (11.5)	38.5 (4.7)	Monthly visits and usual care with medical assistants. 24 contacts.	Medical Assistants and PCP	6 to 8 hours of training to PCPs and lifestyle coaches.	Usual Care	24 months<br><br>F-up: 6,12,18,24 months

Welzel, 2021, Germany, Cluster RCT[40 41]	135	BMI ≥30 kg/m^2, aged between 18 and 60 years old	Acute medical condition that required prioritized treatment.	62.2	43.3 (10.7)	39.0 (6.0)	Training for the GP's through the 5A's tutorial, online. 1 + contacts.	GP's	Short online training of the 5A's approach.	Wait list control	12 months<br><br>F-up: 12 months
Yardley, 2014, UK, RCT[42]	134	BMI 30 ⩾kg/m^2 (or 28 with hypertension, hypercholesterolaemia or diabetes)	Pregnant or breastfeeding, current major health problems, self-reported inability to walk 100 metres.	65	51.2 (13.2)	36 (5.8)	12 week Online self-management programme plus nurse support. Group 1 basic nurse support: 3 sessions over 3 months. 3 contacts. Group 2, regular nurse support 7 contacts either face to face or telephone.	Nurse and online programme	Brief structured training materials accessed on the intervention website to offer help to use the website.	Usual Care	6 months<br><br>F-up: 6 and 12 months

PCP= primary care practitioner, GP= General Practitioner, f-up= follow-up, CVD= cardiovascular disease, TDR = total diet replacement, DPP = Diabetes Prevention Programme, SD = standard deviation, BWMP = behavioural weight management programme, Mas = Medical Assistants, MI = Motivational interviewing, PA = physical activity, H = hours, NR = Not recorded, Min = Minutes

Supplementary Figure 1: Funnel plot of results of weight change from baseline to 12 months

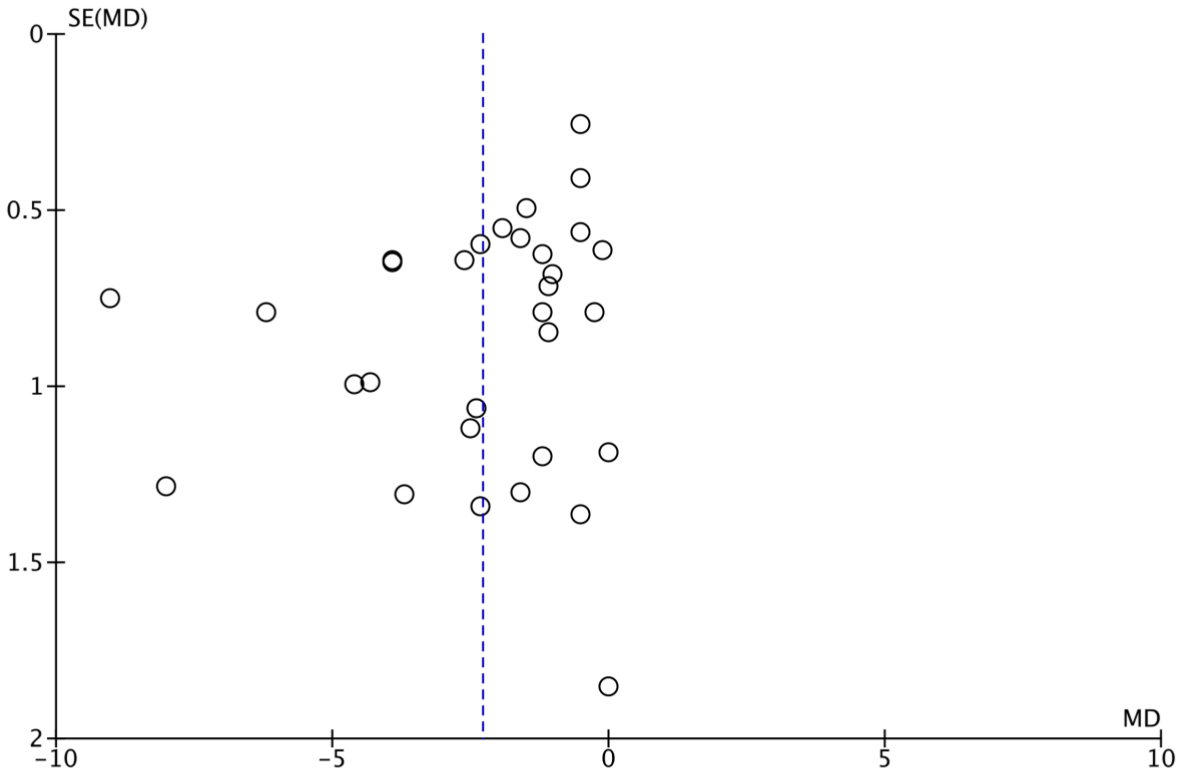

Supplementary Figure 2: Weight Change at last follow-up by provider

Study or Subgroup	Intervention Mean	Intervention SD	Intervention Total	Comparator Mean	Comparator SD	Comparator Total	Weight	Mean Difference IV, Random, 95% CI
**8.1.1 Nurse Led**								
Brautgan–ewe 2020	−1.1	3.2	77	−0.9	3.5	105	3.6%	−0.20 [−1.18, 0.78]
Carrington 2021	−0.9	5.3	118	−1.3	7	122	3.1%	0.40 [−1.17, 1.97]
Little 2017	−3.8	7.4	221	−2.6	9.2	227	3.1%	−1.20 [−2.74, 0.34]
Yardley Basic 2014	−4.3	6.2	44	−2	4.5	22	2.2%	−2.30 [−4.93, 0.33]
Yardley Regular 2014	−2.5	6.5	47	−2	4.5	21	2.1%	−0.50 [−3.18, 2.18]
**Subtotal (95% CI)**			**507**			**497**	**14.1%**	**−0.46 [−1.17, 0.26]**
Heterogeneity: $Tau^2 = 0.03$; $Chi^2 = 4.19$, df = 4 (P = 0.38); $I^2 = 5\%$								
Test for overall effect: Z = 1.26 (P = 0.21)								
**8.1.2 GP Led**								
Christian 2008	−0.2	10.9	141	1.4	10.6	132	2.2%	−1.60 [−4.15, 0.95]
Christian 2011	−1.5	5.3	133	0.1	4	130	3.5%	−1.60 [−2.73, −0.47]
Martin 2008	−0.5	3.3	68	0.07	3.8	69	3.4%	−0.57 [−1.76, 0.62]
Welzel 2021	−2.9	8.3	52	−2.9	11.5	63	1.5%	0.00 [−3.63, 3.63]
**Subtotal (95% CI)**			**394**			**394**	**10.7%**	**−1.11 [−1.87, −0.34]**
Heterogeneity: $Tau^2 = 0.00$; $Chi^2 = 2.01$, df = 3 (P = 0.57); $I^2 = 0\%$								
Test for overall effect: Z = 2.84 (P = 0.005)								
**8.1.3 Non Medical Led**								
Appel 2011 In person support	−5.1	9.2	133	−0.8	8	65	2.3%	−4.30 [−6.80, −1.80]
Appel Remote support 2011	−4.5	8.3	139	−0.8	8	64	2.4%	−3.70 [−6.10, −1.30]
Baer 2020	−3.1	5.3	298	−1.2	8.3	326	3.5%	−1.90 [−2.98, −0.82]
Katzmarzyk 2020	−5.4	11.9	452	−0.9	11.4	351	3.0%	−4.50 [−6.12, −2.88]
Kumanyika 2012	−1.6	5.1	89	−0.6	4.1	98	3.3%	−1.00 [−2.33, 0.33]
Nanchahal 2012	−2.4	5.5	103	−1.3	5	114	3.2%	−1.10 [−2.50, 0.30]
Ross 2012	−1.2	6.6	249	−0.6	4.4	250	3.6%	−0.60 [−1.58, 0.38]
Tsai 2009	−2.3	4.2	22	−1.1	4	25	2.4%	−1.20 [−3.55, 1.15]
Wadden 2011	−2.9	8	131	−1.7	8	130	2.7%	−1.20 [−3.14, 0.74]
**Subtotal (95% CI)**			**1616**			**1423**	**26.4%**	**−2.00 [−2.93, −1.06]**
Heterogeneity: $Tau^2 = 1.31$; $Chi^2 = 25.42$, df = 8 (P = 0.001); $I^2 = 69\%$								
Test for overall effect: Z = 4.18 (P < 0.0001)								
**8.1.4 Other Practitioner**								
Beeken 2017	−2.2	5.8	155	−3	7.2	158	3.2%	0.80 [−0.65, 2.25]
Bennett 2018	−4	5.9	170	−0.1	5.9	167	3.4%	−3.90 [−5.16, −2.64]
Cai 2019	−3.2	3.4	214	−0.03	2.5	208	3.9%	−3.17 [−3.74, −2.60]
Conroy 2014	−1.4	6.8	46	−1.4	3.8	36	2.4%	0.00 [−2.32, 2.32]
Delahanty 2020	−4.6	6.3	70	−2.1	4.7	32	2.5%	−2.50 [−4.70, −0.30]
Delahanty 2020 in person	−4.5	5.5	69	−2.1	4.7	32	2.6%	−2.40 [−4.48, −0.32]
Gomez–Hueglas 2015	0.4	9.5	230	0.9	6.6	176	3.1%	−0.50 [−2.07, 1.07]
Huseinovic 2018	−6.9	7.2	54	−4.6	6.9	56	2.2%	−2.30 [−4.94, 0.34]
Jolly 2011	0.83	5.2	70	1.08	4.9	100	3.1%	−0.25 [−1.80, 1.30]
Lean 2018	−7.6	6.5	129	−2.3	5.2	143	3.2%	−5.30 [−6.71, −3.89]
Logue 2005	−0.4	6.9	329	−0.2	7.7	336	3.5%	−0.20 [−1.31, 0.91]
Phelan 2017	−3.2	5.7	174	−0.9	5.7	193	3.4%	−2.30 [−3.47, −1.13]
Rodriguez–Cristobal 2017	−2.5	7.7	238	−1	5.6	199	3.4%	−1.50 [−2.75, −0.25]
Taheri 2020	−12	9.5	70	−4	5.3	77	2.3%	−8.00 [−10.52, −5.48]
TarragaMarcos 2018 Intensive	−5.6	2.2	60	−1.7	3.1	28	3.3%	−3.90 [−5.18, −2.62]
TarragaMarcos 2018 less intensive	−4.3	1.9	61	−1.7	3.1	27	3.4%	−2.60 [−3.86, −1.34]
**Subtotal (95% CI)**			**2139**			**1968**	**48.8%**	**−2.33 [−3.24, −1.42]**
Heterogeneity: $Tau^2 = 2.78$; $Chi^2 = 105.98$, df = 15 (P < 0.00001); $I^2 = 86\%$								
Test for overall effect: Z = 5.01 (P < 0.00001)								
**Total (95% CI)**			**4656**			**4282**	**100.0%**	**−1.88 [−2.45, −1.30]**
Heterogeneity: $Tau^2 = 2.15$; $Chi^2 = 172.21$, df = 33 (P < 0.00001); $I^2 = 81\%$								
Test for overall effect: Z = 6.39 (P < 0.00001)								
Test for subgroup differences: $Chi^2 = 12.69$, df = 3 (P = 0.005), $I^2 = 76.4\%$								

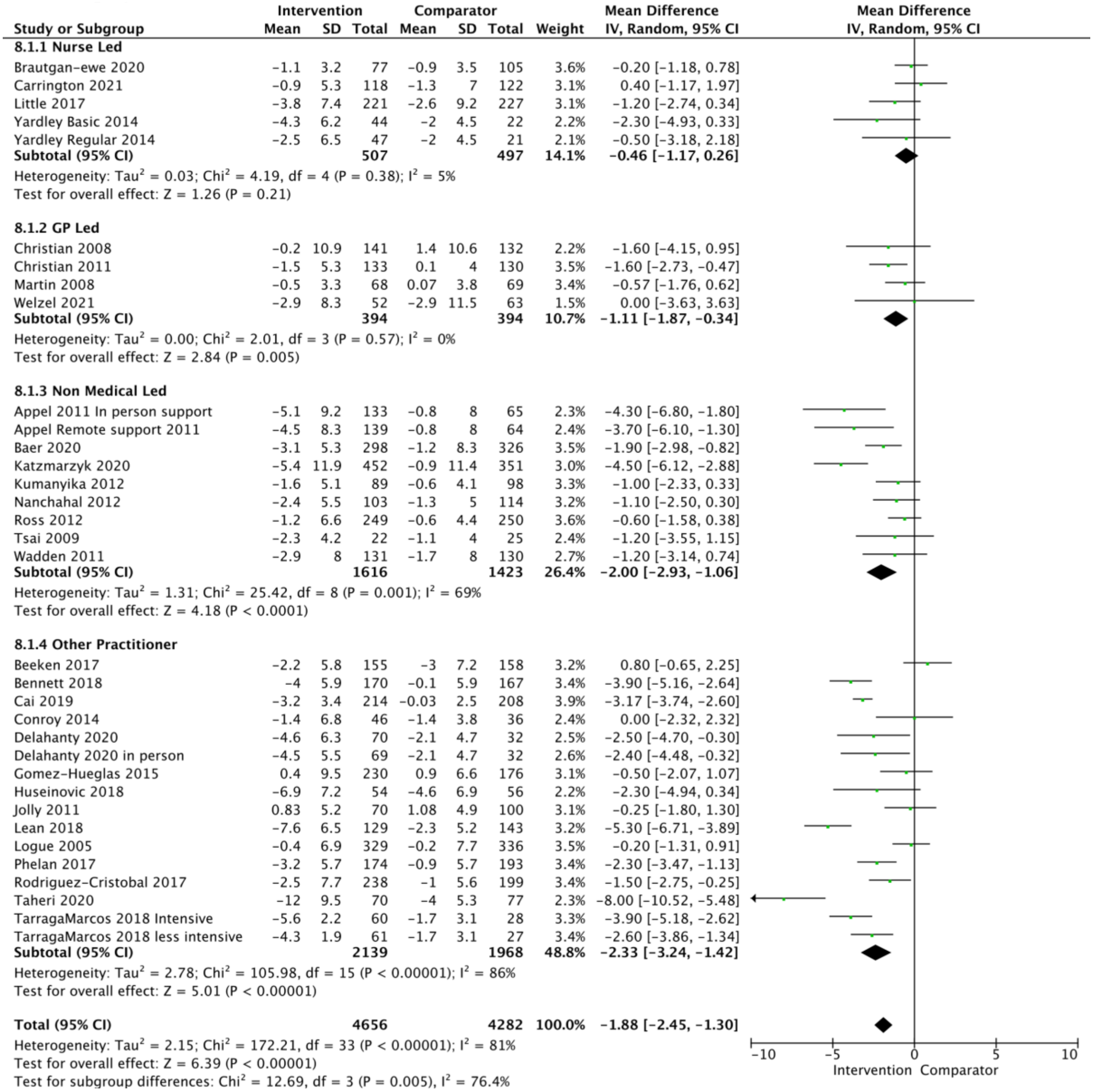

Supplementary Figure 3: Funnel plot of weight change from baseline to last follow-up

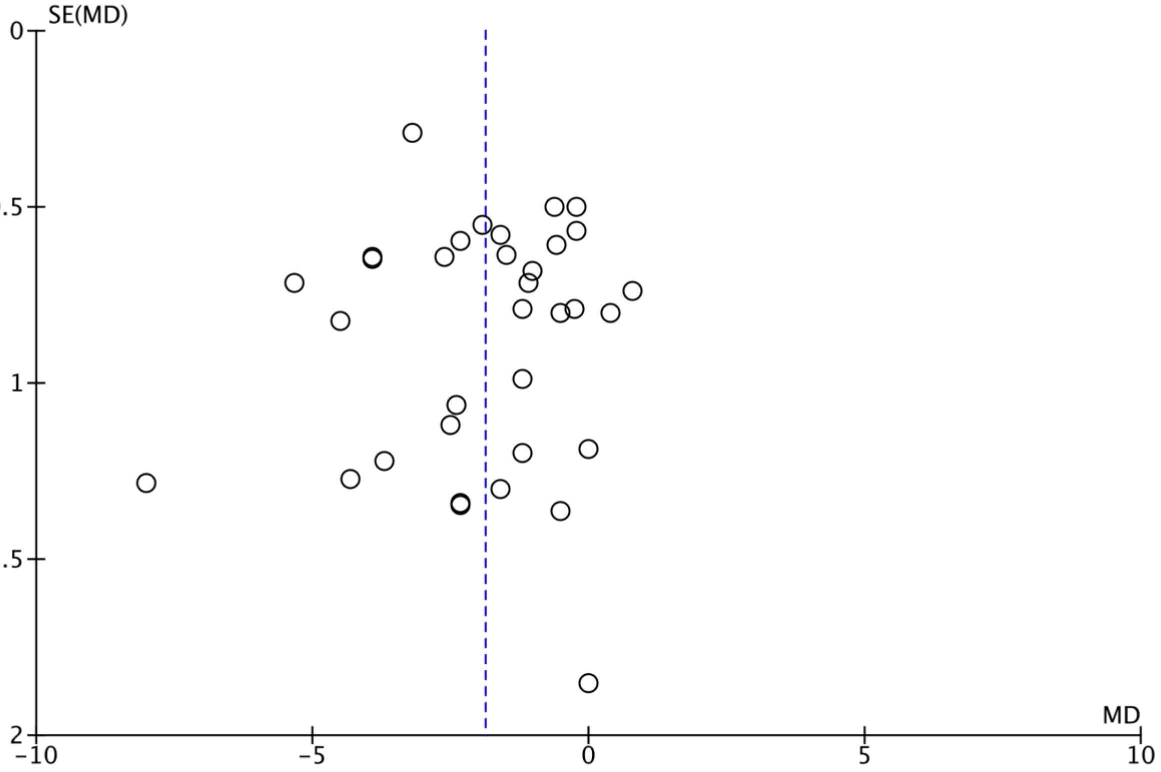

Supplementary Figure 4: Weight change ≥ 24 months follow-up

Study or Subgroup	Intervention Mean	Intervention SD	Intervention Total	Comparator Mean	Comparator SD	Comparator Total	Weight	Mean Difference IV, Random, 95% CI
Appel 2011 In person support	−5.1	9.2	133	−0.8	8	65	5.7%	−4.30 [−6.80, −1.80]
Appel Remote support 2011	−4.5	8.3	139	−0.8	8	64	5.9%	−3.70 [−6.10, −1.30]
Beeken 2017	−2.2	5.8	155	−3	7.2	158	7.4%	0.80 [−0.65, 2.25]
Brautgan-ewe 2020	−1.1	3.2	77	−0.9	3.5	105	8.0%	−0.20 [−1.18, 0.78]
Cai 2019	−3.2	3.4	214	−0.03	2.5	208	8.4%	−3.17 [−3.74, −2.60]
Carrington 2021	−0.9	5.3	118	−1.3	7	122	7.2%	0.40 [−1.17, 1.97]
Gomez-Hueglas 2015	0.4	9.5	230	0.9	6.6	176	7.2%	−0.50 [−2.07, 1.07]
Huseinovic 2018	−6.9	7.2	54	−4.6	6.9	56	5.5%	−2.30 [−4.94, 0.34]
Katzmarzyk 2020	−5.4	11.9	452	−0.9	11.4	351	7.1%	−4.50 [−6.12, −2.88]
Lean 2018	−7.6	6.5	129	−2.3	5.2	143	7.4%	−5.30 [−6.71, −3.89]
Logue 2005	−0.4	6.9	329	−0.2	7.7	336	7.9%	−0.20 [−1.31, 0.91]
Rodriguez-Cristobal 2017	−2.5	7.7	238	−1	5.6	199	7.7%	−1.50 [−2.75, −0.25]
Ross 2012	−1.2	6.6	249	−0.6	4.4	250	8.0%	−0.60 [−1.58, 0.38]
Wadden 2011	−2.9	8	131	−1.7	8	130	6.6%	−1.20 [−3.14, 0.74]
**Total (95% CI)**			**2648**			**2363**	**100.0%**	**−1.79 [−2.81, −0.77]**

Heterogeneity: $Tau^2 = 3.13$; $Chi^2 = 110.08$, df = 13 (P < 0.00001); $I^2 = 88\%$
Test for overall effect: Z = 3.44 (P = 0.0006)

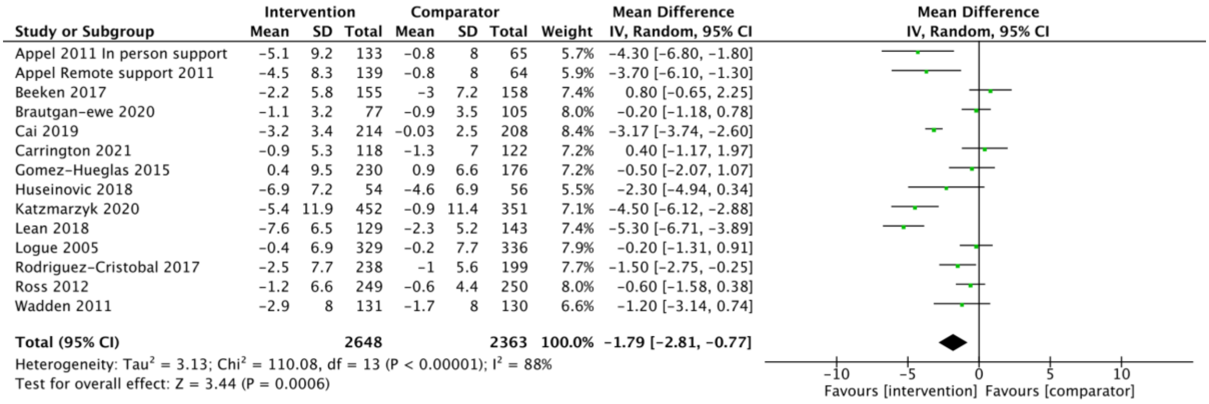

Supplementary Figure 5: Weight change at last follow-up by country

Study or Subgroup	Intervention Mean	Intervention SD	Intervention Total	Comparator Mean	Comparator SD	Comparator Total	Weight	Mean Difference IV, Random, 95% CI
**5.1.1 US**								
Appel 2011 In person support	−5.1	9.2	133	−0.8	8	65	2.7%	−4.30 [−6.80, −1.80]
Appel Remote support 2011	−4.5	8.3	139	−0.8	8	64	2.9%	−3.70 [−6.10, −1.30]
Baer 2020	−3.1	5.3	298	−1.2	8.3	326	4.5%	−1.90 [−2.98, −0.82]
Bennett 2018	−4	5.9	170	−0.1	5.9	167	4.3%	−3.90 [−5.16, −2.64]
Christian 2008	−0.2	10.9	141	1.4	10.6	132	2.7%	−1.60 [−4.15, 0.95]
Christian 2011	−1.5	5.3	133	0.1	4	130	4.4%	−1.60 [−2.73, −0.47]
Conroy 2014	−1.4	6.8	46	−1.4	3.8	36	2.9%	0.00 [−2.32, 2.32]
Delahanty 2020	−4.6	6.3	70	−2.1	4.7	32	3.1%	−2.50 [−4.70, −0.30]
Delahanty 2020 in person	−4.5	5.5	69	−2.1	4.7	32	3.2%	−2.40 [−4.48, −0.32]
Katzmarzyk 2020	−5.4	11.9	452	−0.9	11.4	351	3.8%	−4.50 [−6.12, −2.88]
Kumanyika 2012	−1.6	5.1	89	−0.6	4.1	98	4.2%	−1.00 [−2.33, 0.33]
Logue 2005	−0.4	6.9	329	−0.2	7.7	336	4.5%	−0.20 [−1.31, 0.91]
Martin 2008	−0.5	3.3	68	0.07	3.8	69	4.4%	−0.57 [−1.76, 0.62]
Phelan 2017	−3.2	5.7	174	−0.9	5.7	193	4.4%	−2.30 [−3.47, −1.13]
Tsai 2009	−2.3	4.2	22	−1.1	4	25	2.9%	−1.20 [−3.55, 1.15]
Wadden 2011	−2.9	8	131	−1.7	8	130	3.4%	−1.20 [−3.14, 0.74]
**Subtotal (95% CI)**			**2464**			**2186**	**58.2%**	**−1.99 [−2.69, −1.30]**
Heterogeneity: $Tau^2 = 1.24$; $Chi^2 = 45.47$, df = 15 (P < 0.0001); $I^2 = 67\%$								
Test for overall effect: Z = 5.62 (P < 0.00001)								
**5.1.2 UK**								
Beeken 2017	−2.2	5.8	155	−3	7.2	158	4.0%	0.80 [−0.65, 2.25]
Jolly 2011	0.8	5.2	70	1.1	4.9	100	3.9%	−0.30 [−1.85, 1.25]
Lean 2018	−7.6	6.5	129	−2.3	5.2	143	4.1%	−5.30 [−6.71, −3.89]
Little 2017	−3.8	7.4	221	−2.6	9.2	227	3.9%	−1.20 [−2.74, 0.34]
Nanchahal 2012	−2.4	5.5	103	−1.3	5	114	4.1%	−1.10 [−2.50, 0.30]
Yardley Basic 2014	−4.3	6.2	44	−2	4.5	22	2.6%	−2.30 [−4.93, 0.33]
Yardley Regular 2014	−2.5	6.5	47	−2	4.5	21	2.6%	−0.50 [−3.18, 2.18]
**Subtotal (95% CI)**			**769**			**785**	**25.2%**	**−1.42 [−3.09, 0.25]**
Heterogeneity: $Tau^2 = 4.22$; $Chi^2 = 41.30$, df = 6 (P < 0.00001); $I^2 = 85\%$								
Test for overall effect: Z = 1.67 (P = 0.10)								
**5.1.3 Spain**								
Gomez-Hueglas 2015	0.4	9.5	230	0.9	6.6	176	3.9%	−0.50 [−2.07, 1.07]
Rodriguez-Cristobal 2017	−2.5	7.7	238	−1	5.6	199	4.3%	−1.50 [−2.75, −0.25]
TarragaMarcos 2018 Intensive	−5.6	2.2	60	−1.7	3.1	28	4.3%	−3.90 [−5.18, −2.62]
TarragaMarcos 2018 less intensive	−4.3	1.9	61	−1.7	3.1	27	4.3%	−2.60 [−3.86, −1.34]
**Subtotal (95% CI)**			**589**			**430**	**16.7%**	**−2.17 [−3.54, −0.79]**
Heterogeneity: $Tau^2 = 1.51$; $Chi^2 = 12.88$, df = 3 (P = 0.005); $I^2 = 77\%$								
Test for overall effect: Z = 3.08 (P = 0.002)								
**Total (95% CI)**			**3822**			**3401**	**100.0%**	**−1.89 [−2.48, −1.29]**

Heterogeneity: $Tau^2 = 1.76$; $Chi^2 = 102.51$, df = 26 (P < 0.00001); $I^2 = 75\%$
Test for overall effect: Z = 6.20 (P < 0.00001)
Test for subgroup differences: $Chi^2 = 0.49$, df = 2 (P = 0.78), $I^2 = 0\%$

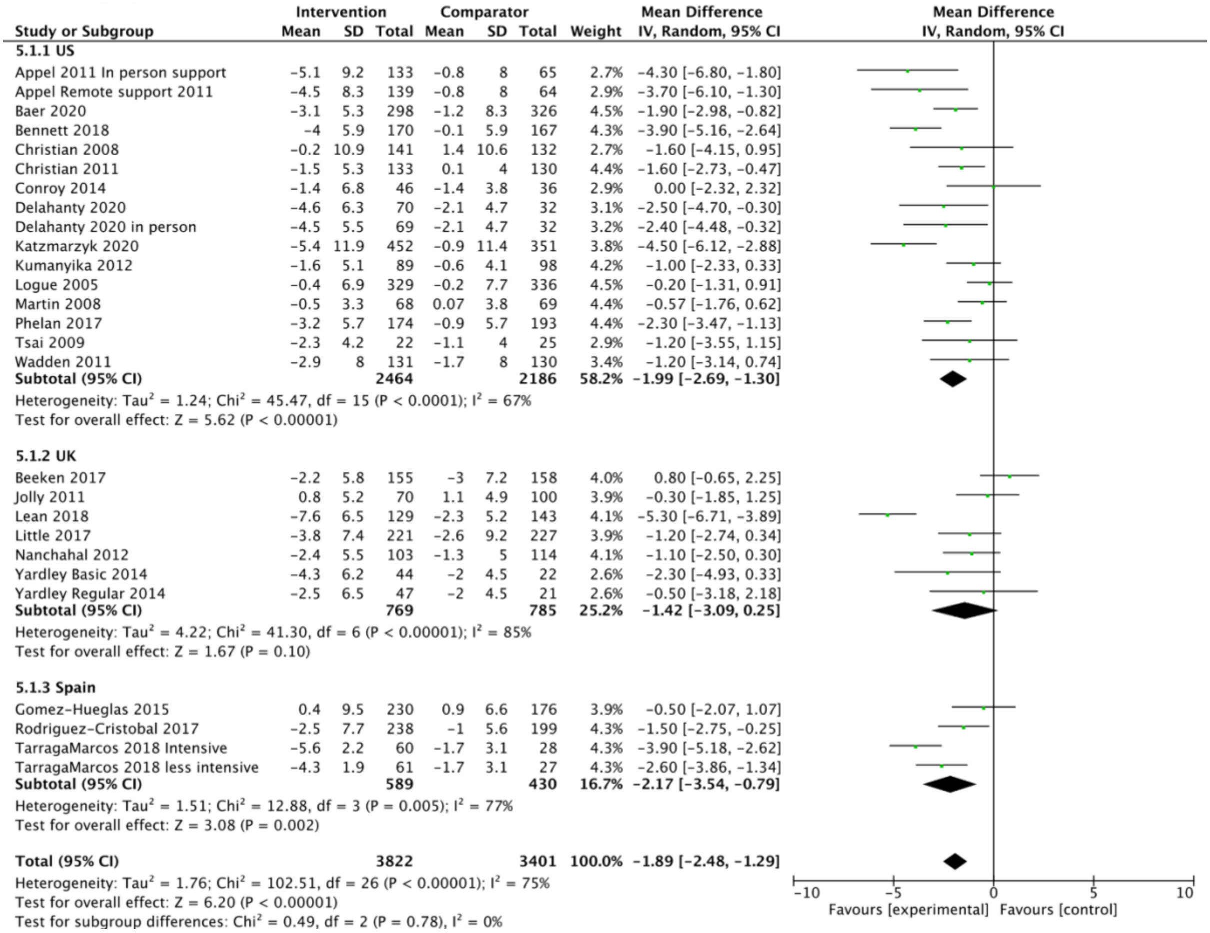

Supplementary Figure 6: Weight change at last follow-up by the number of contacts

Study or Subgroup	Intervention Mean	Intervention SD	Intervention Total	Comparator Mean	Comparator SD	Comparator Total	Weight	Mean Difference IV, Random, 95% CI
**9.1.1 11 or less contacts**								
Beeken 2017	−2.2	5.8	155	−3	7.2	158	3.2%	0.80 [−0.65, 2.25]
Brautgan−ewe 2020	−1.1	3.2	77	−0.9	3.5	105	3.6%	−0.20 [−1.18, 0.78]
Carrington 2021	−0.9	5.3	118	−1.3	7	122	3.1%	0.40 [−1.17, 1.97]
Christian 2008	−0.2	10.9	141	1.4	10.6	132	2.2%	−1.60 [−4.15, 0.95]
Christian 2011	−1.5	5.3	133	0.1	4	130	3.5%	−1.60 [−2.73, −0.47]
Huseinovic 2018	−6.9	7.2	54	−4.6	6.9	56	2.2%	−2.30 [−4.94, 0.34]
Little 2017	−3.8	7.4	221	−2.6	9.2	227	3.1%	−1.20 [−2.74, 0.34]
Martin 2008	−0.5	3.3	68	0.07	3.8	69	3.4%	−0.57 [−1.76, 0.62]
Welzel 2021	−2.9	8.3	52	−2.9	11.5	63	1.5%	0.00 [−3.63, 3.63]
Yardley Basic 2014	−4.3	6.2	44	−2	4.5	22	2.2%	−2.30 [−4.93, 0.33]
Yardley Regular 2014	−2.5	6.5	47	−2	4.5	21	2.1%	−0.50 [−3.18, 2.18]
**Subtotal (95% CI)**			**1110**			**1105**	**30.1%**	**−0.65 [−1.23, −0.08]**
Heterogeneity: $Tau^2 = 0.22$; $Chi^2 = 13.27$, df = 10 (P = 0.21); $I^2 = 25\%$								
Test for overall effect: Z = 2.22 (P = 0.03)								
**9.1.2 12 or more contacts**								
Appel 2011 In person support	−5.1	9.2	133	−0.8	8	65	2.3%	−4.30 [−6.80, −1.80]
Appel Remote support 2011	−4.5	8.3	139	−0.8	8	64	2.4%	−3.70 [−6.10, −1.30]
Baer 2020	−3.1	5.3	298	−1.2	8.3	326	3.5%	−1.90 [−2.98, −0.82]
Bennett 2018	−4	5.9	170	−0.1	5.9	167	3.4%	−3.90 [−5.16, −2.64]
Cai 2019	−3.2	3.4	214	−0.03	2.5	208	3.9%	−3.17 [−3.74, −2.60]
Conroy 2014	−1.4	6.8	46	−1.4	3.8	36	2.4%	0.00 [−2.32, 2.32]
Delahanty 2020	−4.6	6.3	70	−2.1	4.7	32	2.5%	−2.50 [−4.70, −0.30]
Delahanty 2020 in person	−4.5	5.5	69	−2.1	4.7	32	2.6%	−2.40 [−4.48, −0.32]
Gomez−Hueglas 2015	0.4	9.5	230	0.9	6.6	176	3.1%	−0.50 [−2.07, 1.07]
Jolly 2011	0.83	5.2	70	1.08	4.9	100	3.1%	−0.25 [−1.80, 1.30]
Katzmarzyk 2020	−5.4	11.9	452	−0.9	11.4	351	3.0%	−4.50 [−6.12, −2.88]
Kumanyika 2012	−1.6	5.1	89	−0.6	4.1	98	3.3%	−1.00 [−2.33, 0.33]
Lean 2018	−7.6	6.5	129	−2.3	5.2	143	3.2%	−5.30 [−6.71, −3.89]
Logue 2005	−0.4	6.9	329	−0.2	7.7	336	3.5%	−0.20 [−1.31, 0.91]
Nanchahal 2012	−2.4	5.5	103	−1.3	5	114	3.2%	−1.10 [−2.50, 0.30]
Phelan 2017	−3.2	5.7	174	−0.9	5.7	193	3.4%	−2.30 [−3.47, −1.13]
Rodriguez−Cristobal 2017	−2.5	7.7	238	−1	5.6	199	3.4%	−1.50 [−2.75, −0.25]
Ross 2012	−1.2	6.6	249	−0.6	4.4	250	3.6%	−0.60 [−1.58, 0.38]
Taheri 2020	−12	9.5	70	−4	5.3	77	2.3%	−8.00 [−10.52, −5.48]
TarragaMarcos 2018 Intensive	−5.6	2.2	60	−1.7	3.1	28	3.3%	−3.90 [−5.18, −2.62]
TarragaMarcos 2018 less intensive	−4.3	1.9	61	−1.7	3.1	27	3.4%	−2.60 [−3.86, −1.34]
Tsai 2009	−2.3	4.2	22	−1.1	4	25	2.4%	−1.20 [−3.55, 1.15]
Wadden 2011	−2.9	8	131	−1.7	8	130	2.7%	−1.20 [−3.14, 0.74]
**Subtotal (95% CI)**			**3546**			**3177**	**69.9%**	**−2.36 [−3.04, −1.68]**
Heterogeneity: $Tau^2 = 2.11$; $Chi^2 = 120.65$, df = 22 (P < 0.00001); $I^2 = 82\%$								
Test for overall effect: Z = 6.78 (P < 0.00001)								
**Total (95% CI)**			**4656**			**4282**	**100.0%**	**−1.88 [−2.45, −1.30]**
Heterogeneity: $Tau^2 = 2.15$; $Chi^2 = 172.21$, df = 33 (P < 0.00001); $I^2 = 81\%$								
Test for overall effect: Z = 6.39 (P < 0.00001)								
Test for subgroup differences: $Chi^2 = 14.00$, df = 1 (P = 0.0002), $I^2 = 92.9\%$								

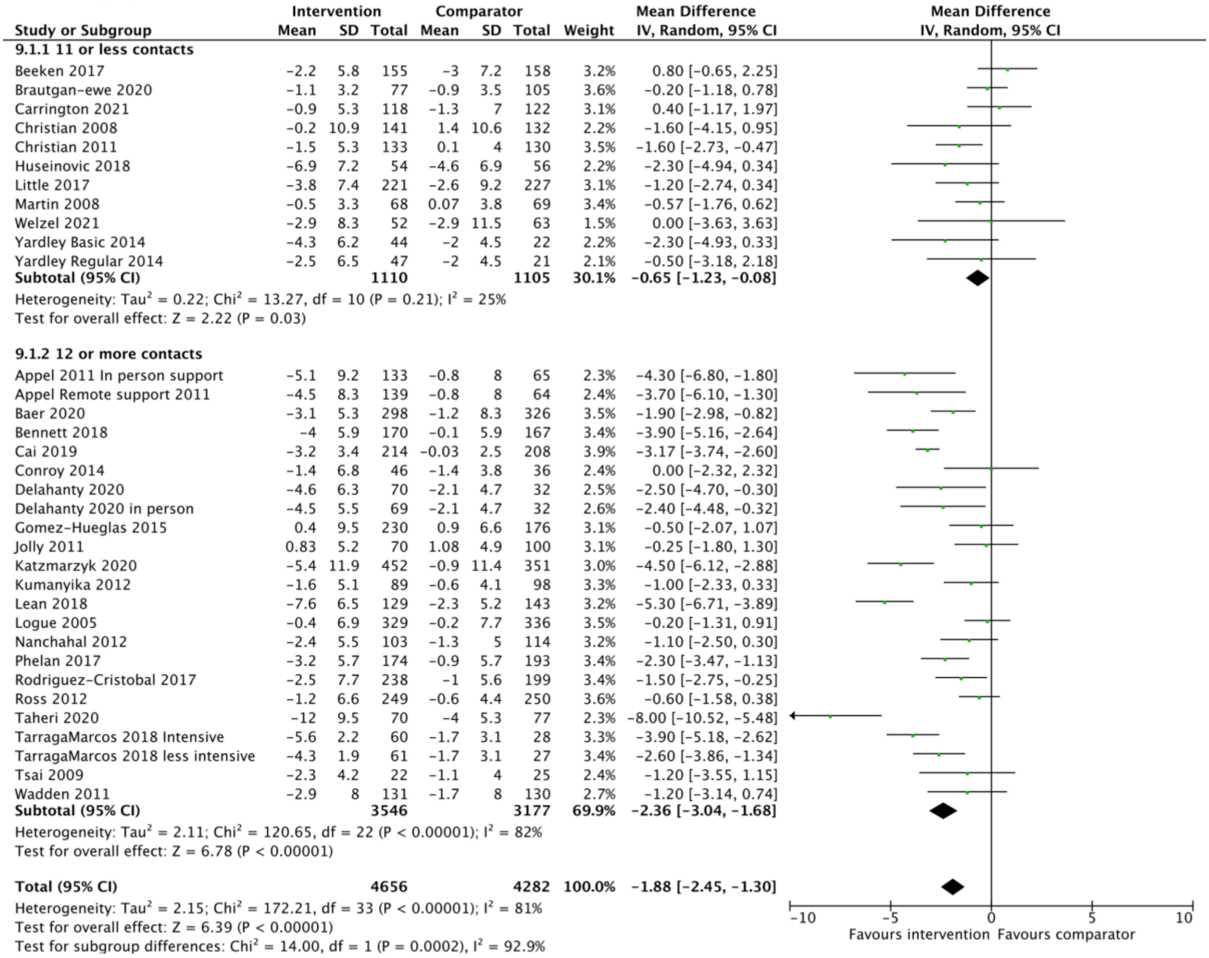

Supplementary figure 7: Risk of bias category and weight change at 12 months

Study or Subgroup	Intervention Mean	Intervention SD	Intervention Total	Comparator Mean	Comparator SD	Comparator Total	Weight	Mean Difference IV, Random, 95% CI
**6.1.1 High Risk of Bias**								
Baer 2020	−3.1	5.3	298	−1.2	8.3	326	3.6%	−1.90 [−2.98, −0.82]
Christian 2011	−1.5	5.3	133	0.1	4	130	3.6%	−1.60 [−2.73, −0.47]
Katzmarzyk 2020	−7.2	11.9	452	−1	10.4	351	3.4%	−6.20 [−7.75, −4.65]
Lean 2018	−10	8	137	−1	3.7	148	3.4%	−9.00 [−10.47, −7.53]
Martin 2008	−1.4	3.7	68	−0.2	3.6	69	3.6%	−1.20 [−2.42, 0.02]
Nanchahal 2012	−2.4	5.5	103	−1.3	5	114	3.4%	−1.10 [−2.50, 0.30]
Phelan 2017	−3.2	5.7	174	−0.9	5.7	193	3.6%	−2.30 [−3.47, −1.13]
Rodriguez−Cristobal 2017	−1.8	6.7	283	−1.3	1.7	302	3.8%	−0.50 [−1.30, 0.30]
Welzel 2021	−2.9	8.3	52	−2.9	11.5	63	2.0%	0.00 [−3.63, 3.63]
**Subtotal (95% CI)**			**1700**			**1696**	**30.4%**	**−2.70 [−4.42, −0.98]**
Heterogeneity: $Tau^2 = 6.32$; $Chi^2 = 133.28$, df = 8 (P < 0.00001); $I^2 = 94\%$								
Test for overall effect: Z = 3.07 (P = 0.002)								
**6.1.2 Low Risk of Bias**								
Appel 2011 In person support	−5.4	7.7	123	−1.1	5.2	54	3.1%	−4.30 [−6.24, −2.36]
Appel Remote support 2011	−5.7	7.8	124	−1.1	5.2	54	3.1%	−4.60 [−6.55, −2.65]
Beeken 2017	−2.4	5.5	143	−2.3	5	149	3.6%	−0.10 [−1.31, 1.11]
Bennett 2018	−4	5.9	170	−0.1	5.9	167	3.5%	−3.90 [−5.16, −2.64]
Carrington 2021	−0.9	4.3	126	−0.4	4.6	122	3.6%	−0.50 [−1.61, 0.61]
Jolly 2011	0.83	5.2	70	1.08	4.9	100	3.4%	−0.25 [−1.80, 1.30]
Kumanyika 2012	−1.6	5.1	89	−0.6	4.1	98	3.5%	−1.00 [−2.33, 0.33]
Ross 2012	−2.4	5.4	249	−0.9	5.6	241	3.7%	−1.50 [−2.47, −0.53]
Taheri 2020	−12	9.5	70	−4	5.3	77	2.7%	−8.00 [−10.52, −5.48]
Wadden 2011	−3.4	6.9	131	−2.3	6.8	130	3.3%	−1.10 [−2.76, 0.56]
**Subtotal (95% CI)**			**1295**			**1192**	**33.4%**	**−2.34 [−3.58, −1.10]**
Heterogeneity: $Tau^2 = 3.34$; $Chi^2 = 67.31$, df = 9 (P < 0.00001); $I^2 = 87\%$								
Test for overall effect: Z = 3.71 (P = 0.0002)								
**6.1.3 Unclear Risk of Bias**								
Christian 2008	−0.2	10.9	141	1.4	10.6	132	2.6%	−1.60 [−4.15, 0.95]
Conroy 2014	−1.4	6.8	46	−1.4	3.8	36	2.8%	0.00 [−2.32, 2.32]
Delahanty 2020	−4.6	6.3	70	−2.1	4.7	32	2.9%	−2.50 [−4.70, −0.30]
Delahanty 2020 in person	−4.5	5.5	69	−2.1	4.7	32	3.0%	−2.40 [−4.48, −0.32]
Huseinovic 2018	−9.3	4.8	44	−5.6	7.3	45	2.6%	−3.70 [−6.26, −1.14]
Little 2017	−3.8	7.4	221	−2.6	9.2	227	3.4%	−1.20 [−2.74, 0.34]
Logue 2005	−1.4	3.2	329	−0.9	3.4	336	3.9%	−0.50 [−1.00, 0.00]
TarragaMarcos 2018 Intensive	−5.6	2.2	60	−1.7	3.1	28	3.5%	−3.90 [−5.18, −2.62]
TarragaMarcos 2018 less intensive	−4.3	1.9	61	−1.7	3.1	27	3.5%	−2.60 [−3.86, −1.34]
Tsai 2009	−2.3	4.2	22	−1.1	4	25	2.8%	−1.20 [−3.55, 1.15]
Yardley Basic 2014	−4.3	6.2	44	−2	4.5	22	2.6%	−2.30 [−4.93, 0.33]
Yardley Regular 2014	−2.5	6.5	47	−2	4.5	21	2.6%	−0.50 [−3.18, 2.18]
**Subtotal (95% CI)**			**1154**			**963**	**36.2%**	**−1.87 [−2.77, −0.98]**
Heterogeneity: $Tau^2 = 1.50$; $Chi^2 = 37.55$, df = 11 (P < 0.0001); $I^2 = 71\%$								
Test for overall effect: Z = 4.11 (P < 0.0001)								
**Total (95% CI)**			**4149**			**3851**	**100.0%**	**−2.29 [−3.00, −1.57]**
Heterogeneity: $Tau^2 = 3.38$; $Chi^2 = 249.29$, df = 30 (P < 0.00001); $I^2 = 88\%$								
Test for overall effect: Z = 6.23 (P < 0.00001)								
Test for subgroup differences: $Chi^2 = 0.85$, df = 2 (P = 0.65), $I^2 = 0\%$								

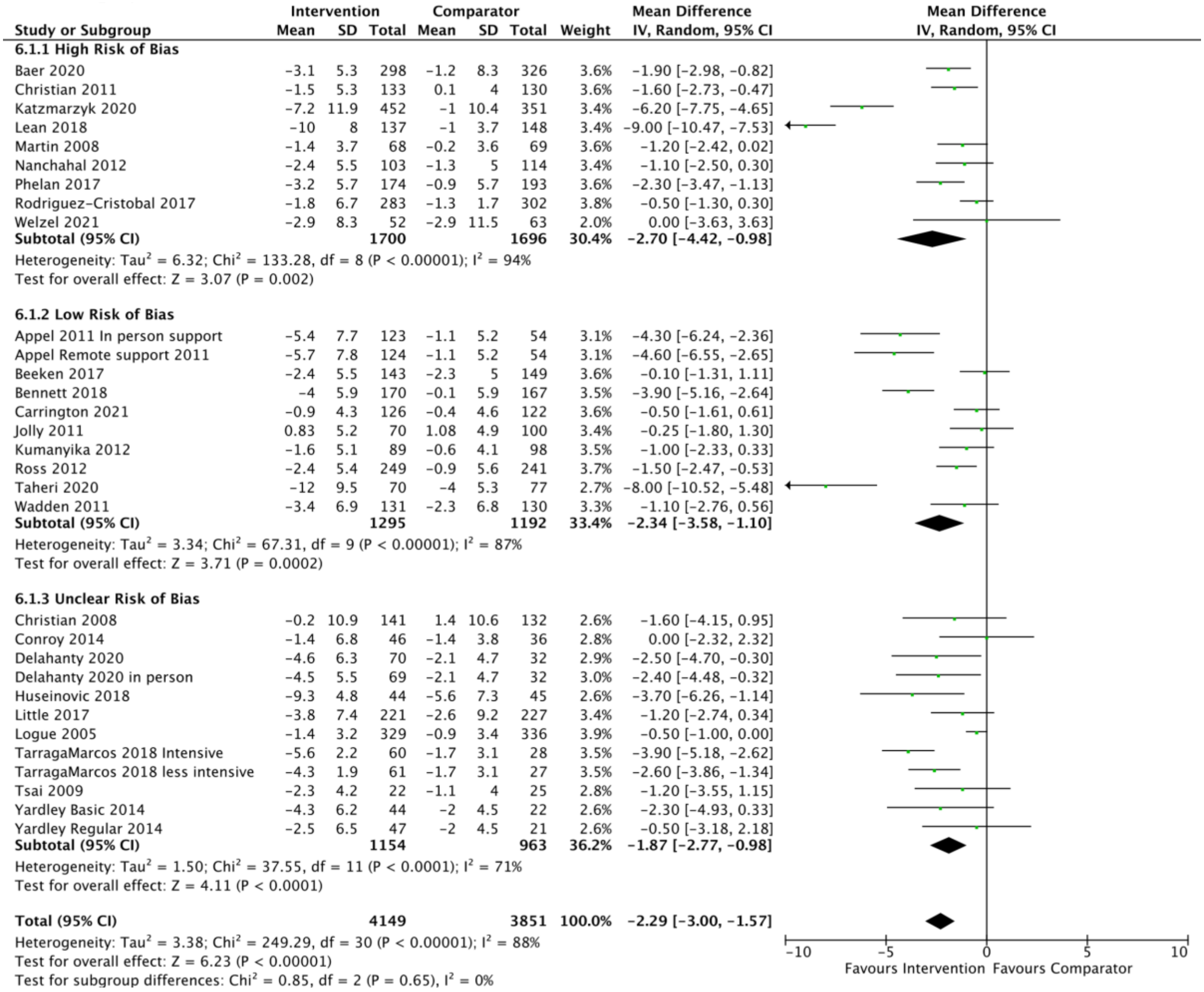

Meta-regression code

```
//Gemma Taylor, March.21.2022

///Import data
import excel "/Volumes/gmjm20/Projects/Claire weight/Data set for Gem for meta regression.xlsx", sheet("Sheet2") firstrow clear

//Convert 95%CI to SE
gen diff= ( Upper95CI-Lower95Ci)
gen se= diff / 3.92
replace se = abs(se)

//Conduct univariate meta-regression to determine the association between i) contacts, and ii) provider on study effect size
foreach i in Numberofsessions Whodeliveredtheintervention {
metareg Meandifference `i', wsse(se)
regsave `i' using "/Volumes/gmjm20/Projects/Claire weight/working_data/analysis_univariate_`i'", replace ci pval cmdline
}

//Conduct multivariable meta-regression to determine the association between contacts and provider on study effect size
metareg Meandifference Numberofsessions Who , wsse(se)
regsave using "/Volumes/gmjm20/Projects/Claire weight/working_data/analysis_multivariable", replace ci pval cmdline

//Append regsave files to create excel table
use "/Volumes/gmjm20/Projects/Claire weight/working_data/analysis_univariate_Numberofsessions",clear
gen analysis="Univariate association between number of sessions on mean difference"

append using "/Volumes/gmjm20/Projects/Claire weight/working_data/analysis_univariate_Who"
replace analysis="Univariate association between who on mean difference" if analysis==""

append using "/Volumes/gmjm20/Projects/Claire weight/working_data/analysis_multivariable"
replace analysis="Multivariate association between number of sessions and who on mean difference" if analysis==""

order analysis var coef ci_lower ci_upper stderr pval N
duplicates drop
drop if var =="_cons"
rename var Variable
rename coef Regression_coefficient
```

```
rename stderr SE
rename analysis Analysis_description
rename pval P_value
rename ci_lower CI_lower
rename ci_upper CI_upper

//round to two decimal places
foreach i in Regression_coefficient CI_lower CI_upper SE P_value {
format `i' %10.2f
}

save "/Volumes/gmjm20/Projects/Claire weight/Metareg_output_2022.03.21", replace
export excel using "/Volumes/gmjm20/Projects/Claire weight/Metareg_output_20220321", sheetreplace firstrow(variables)
```

References

1. Appel LJ, Clark JM, Yeh H-C, et al. Comparative effectiveness of weight-loss interventions in clinical practice. *New England Journal of Medicine* 2011;365(21):1959-68. doi: doi:10.1056/NEJMoa1108660
2. Baer HJ, Rozenblum R, De La Cruz BA, et al. Effect of an Online Weight Management Program Integrated With Population Health Management on Weight Change: A Randomized Clinical Trial. *Jama* 2020;324(17):1737-46. doi: 10.1001/jama.2020.18977 [published Online First: 2020/11/04]
3. Beeken RJ, Leurent B, Vickerstaff V, et al. A brief intervention for weight control based on habit-formation theory delivered through primary care: results from a randomised controlled trial. *International Journal of Obesity* 2017;41(2):246-54. doi: 10.1038/ijo.2016.206
4. Bennett GG, Steinberg D, Askew S, et al. Effectiveness of an App and Provider Counseling for Obesity Treatment in Primary Care. *Am J Prev Med* 2018;55(6):777-86. doi: 10.1016/j.amepre.2018.07.005 [published Online First: 2018/10/27]
5. Bräutigam-Ewe M, Lydell M, Bergh H, et al. Two-year weight, risk and health factor outcomes of a weight-reduction intervention programme: Primary prevention for overweight in a multicentre primary healthcare setting. *Scand J Prim Health Care* 2020;38(2):192-200. doi: 10.1080/02813432.2020.1753379 [published Online First: 2020/05/05]
6. Cai R, Chao J, Li D, et al. Effect of community-based lifestyle interventions on weight loss and cardiometabolic risk factors in obese elderly in China: A randomized controlled trial. *Exp Gerontol* 2019;128:110749. doi: 10.1016/j.exger.2019.110749 [published Online First: 2019/10/24]
7. Carrington MJ, Zimmet PZ. Nurse co-ordinated health and lifestyle modification for reducing multiple cardio-metabolic risk factors in regional adults: outcomes from the MODERN randomized controlled trial. *Eur J Cardiovasc Nurs* 2021 doi: 10.1093/eurjcn/zvab042 [published Online First: 2021/04/27]
8. Christian JG, Bessesen DH, Byers TE, et al. Clinic-based support to help overweight patients with type 2 diabetes increase physical activity and lose weight. *Arch Intern Med* 2008;168(2):141-6. doi: 10.1001/archinternmed.2007.13 [published Online First: 2008/01/30]

9. Christian JG, Byers TE, Christian KK, et al. A computer support program that helps clinicians provide patients with metabolic syndrome tailored counseling to promote weight loss. *J Am Diet Assoc* 2011;111(1):75-83. doi: 10.1016/j.jada.2010.10.006 [published Online First: 2010/12/28]
10. Conroy MB, Sward KL, Spadaro KC, et al. Effectiveness of a physical activity and weight loss intervention for middle-aged women: healthy bodies, healthy hearts randomized trial. *J GEN INTERN MED* 2015;30(2):207-13. doi: 10.1007/s11606-014-3077-5 [published Online First: 2014/11/14]
11. Delahanty LM, Chang Y, Levy DE, et al. Design and participant characteristics of a primary care adaptation of the Look AHEAD Lifestyle Intervention for weight loss in type 2 diabetes: The REAL HEALTH-diabetes study. *Contemp Clin Trials* 2018;71:9-17. doi: 10.1016/j.cct.2018.05.018 [published Online First: 2018/05/29]
12. Fernández-Ruiz VE, Armero-Barranco D, Paniagua-Urbano JA, et al. Short-medium-long-term efficacy of interdisciplinary intervention against overweight and obesity: Randomized controlled clinical trial. *Int J Nurs Pract* 2018;24(6):e12690. doi: 10.1111/ijn.12690 [published Online First: 2018/08/16]
13. Fernández-Ruiz VE, Ramos-Morcillo AJ, Solé-Agustí M, et al. Effectiveness of an Interdisciplinary Program Performed on Obese People Regarding Nutritional Habits and Metabolic Comorbidity: A Randomized Controlled Clinical Trial. *Int J Environ Res Public Health* 2020;17(1) doi: 10.3390/ijerph17010336 [published Online First: 2020/01/18]
14. Gomez-Huelgas R, Jansen-Chaparro S, Baca-Osorio AJ, et al. Effects of a long-term lifestyle intervention program with Mediterranean diet and exercise for the management of patients with metabolic syndrome in a primary care setting. *Eur J Intern Med* 2015;26(5):317-23. doi: 10.1016/j.ejim.2015.04.007 [published Online First: 2015/04/25]
15. Huseinovic E, Bertz F, Brekke HK, et al. Two-year follow-up of a postpartum weight loss intervention: Results from a randomized controlled trial. *Matern Child Nutr* 2018;14(2):e12539. doi: 10.1111/mcn.12539 [published Online First: 2017/10/07]
16. Huseinovic E, Bertz F, Leu Agelii M, et al. Effectiveness of a weight loss intervention in postpartum women: results from a randomized controlled trial in primary health care. *Am J Clin Nutr* 2016;104(2):362-70. doi: 10.3945/ajcn.116.135673 [published Online First: 2016/07/15]
17. Jolly K, Lewis A, Beach J, et al. Comparison of range of commercial or primary care led weight reduction programmes with minimal intervention control for weight loss in obesity: Lighten Up randomised controlled trial. *BMJ* 2011;343 doi: 10.1136/bmj.d6500
18. Jolly K, Lewis A, Beach J, et al. A randomised controlled trial to compare a range of commercial or primary care led weight reduction programmes with a aminimal intervention control for weight loss in obesity: the Lighten up trial. *British Medical Journal* 2011
19. Kanke S, Kawai T, Takasawa N, et al. Interventions for body weight reduction in obese patients during short consultations: an open-label randomized controlled trial in the Japanese primary care setting. *Asia Pac Fam Med* 2015;14(1):5-5. doi: 10.1186/s12930-015-0022-7
20. Katzmarzyk PT, Martin CK, Newton RL, Jr., et al. Promoting Successful Weight Loss in Primary Care in Louisiana (PROPEL): Rationale, design and baseline characteristics. *Contemp Clin Trials* 2018;67:1-10. doi: 10.1016/j.cct.2018.02.002 [published Online First: 2018/02/07]

21. Katzmarzyk PT, Martin CK, Newton RL, Jr., et al. Weight Loss in Underserved Patients - A Cluster-Randomized Trial. *N Engl J Med* 2020;383(10):909-18. doi: 10.1056/NEJMoa2007448 [published Online First: 2020/09/03]
22. Kumanyika SK, Morales KH, Allison KC, et al. Two-Year Results of Think Health! ¡Vive Saludable!: A Primary Care Weight-Management Trial. *Obesity (Silver Spring)* 2018;26(9):1412-21. doi: 10.1002/oby.22258 [published Online First: 2018/08/31]
23. Kumanyika SK, Fassbender JE, Sarwer DB, et al. One-year results of the Think Health! study of weight management in primary care practices. *Obesity (Silver Spring)* 2012;20(6):1249-57. doi: 10.1038/oby.2011.329 [published Online First: 2011/11/05]
24. Lean ME, Leslie WS, Barnes AC, et al. Primary care-led weight management for remission of type 2 diabetes (DiRECT): an open-label, cluster-randomised trial. *Lancet* 2018;391(10120):541-51. doi: 10.1016/s0140-6736(17)33102-1 [published Online First: 2017/12/10]
25. Lean MEJ, Leslie WS, Barnes AC, et al. Durability of a primary care-led weight-management intervention for remission of type 2 diabetes: 2-year results of the DiRECT open-label, cluster-randomised trial. *Lancet Diabetes Endocrinol* 2019;7(5):344-55. doi: 10.1016/s2213-8587(19)30068-3 [published Online First: 2019/03/11]
26. Little P, Stuart B, Hobbs R, et al. Randomised controlled trial and economic analysis of an internet-based weight management programme: POWeR+ (Positive Online Weight Reduction). *Health Technol Assess* 2017;21(4)
27. Little P, Stuart B, Hobbs R, et al. An internet-based intervention with brief nurse support to manage obesity in primary care (POWeR+): a pragmatic, parallel-group, randomised controlled trial. *The Lancet Diabetes & Endocrinology* 2016;4(10):821-28.
28. Logue E, Sutton K, Jarjoura D, et al. Transtheoretical model-chronic disease care for obesity in primary care: a randomized trial. *Obes Res* 2005;13(5):917-27. doi: 10.1038/oby.2005.106 [published Online First: 2005/05/28]
29. Martin PD, Dutton GR, Rhode PC, et al. Weight loss maintenance following a primary care intervention for low-income minority women. *Obesity (Silver Spring)* 2008;16(11):2462-7. doi: 10.1038/oby.2008.399 [published Online First: 2008/09/13]
30. Moore H, Summerbell C, Greenwood D, et al. Improving management of obesity in primary care: cluster randomised trial. *BMJ* 2003;327(7423):1085. doi: 10.1136/bmj.327.7423.1085
31. Nanchahal K, Power T, Holdsworth E, et al. A pragmatic randomised controlled trial in primary care of the Camden Weight Loss (CAMWEL) programme. *BMJ Open* 2012;2:e000793.
32. Phelan S, Hagobian T, Brannen A, et al. Effect of an Internet-Based Program on Weight Loss for Low-Income Postpartum Women: A Randomized Clinical Trial. *JAMA* 2017;317(23):2381-91. doi: 10.1001/jama.2017.7119
33. Pritchard DA, Hyndman J, Taba F. Nutritional counselling in general practice: a cost effective analysis. *Journal of epidemiology and community health* 1999;53(5):311-16. doi: 10.1136/jech.53.5.311
34. Rodriguez-Cristobal JJ, Alonso-Villaverde C, Panisello JM, et al. Effectiveness of a motivational intervention on overweight/obese patients in the primary healthcare: a cluster randomized trial. *BMC Fam Pract* 2017;18(1):74. doi: 10.1186/s12875-017-0644-y [published Online First: 2017/06/22]
35. Ross R, Lam M, Blair SN, et al. Trial of prevention and reduction of obesity through active living in clinical settings: a randomized controlled trial. *Arch Intern Med*

2012;172(5):414-24. doi: 10.1001/archinternmed.2011.1972 [published Online First: 2012/03/01]
36. Taheri S, Zaghloul H, Chagoury O, et al. Effect of intensive lifestyle intervention on bodyweight and glycaemia in early type 2 diabetes (DIADEM-I): an open-label, parallel-group, randomised controlled trial. *The Lancet Diabetes & Endocrinology* 2020;8(6):477-89. doi: https://doi.org/10.1016/S2213-8587(20)30117-0
37. Tárraga Marcos ML, Panisello Royo JM, Carbayo Herencia JA, et al. [Analysis of clinical relevance applied to 3methods of reducing weight in overweight or obesity followed-up for one year]. *Hipertens Riesgo Vasc* 2018;35(1):5-14. doi: 10.1016/j.hipert.2017.06.004 [published Online First: 2017/09/17]
38. Tsai AG, Wadden TA, Rogers MA, et al. A primary care intervention for weight loss: results of a randomized controlled pilot study. *Obesity (Silver Spring)* 2010;18(8):1614-8. doi: 10.1038/oby.2009.457 [published Online First: 2009/12/19]
39. Wadden TA, Neiberg RH, Wing RR, et al. Four-Year Weight Losses in the Look AHEAD Study: Factors associated with Long-Term Success. *Obesity* 2011;19(10):1987-98.
40. Welzel FD, Bär J, Stein J, et al. Using a brief web-based 5A intervention to improve weight management in primary care: results of a cluster-randomized controlled trial. *BMC Fam Pract* 2021;22(1):61. doi: 10.1186/s12875-021-01404-0 [published Online First: 2021/04/03]
41. Welzel FD, Stein J, Pabst A, et al. Five A's counseling in weight management of obese patients in primary care: a cluster-randomized controlled trial (INTERACT). *BMC Fam Pract* 2018;19(1):97. doi: 10.1186/s12875-018-0785-7 [published Online First: 2018/06/25]
42. Yardley L, Ware LJ, Smith ER, et al. Randomised controlled feasibility trial of a web-based weight management intervention with nurse support for obese patients in primary care. *Int J Behav Nutr Phys Act* 2014;11:67. doi: 10.1186/1479-5868-11-67 [published Online First: 2014/06/03]
43. Wadden TA, Walsh OA, Berkowitz RI, et al. Intensive Behavioral Therapy for Obesity Combined with Liraglutide 3.0 mg: A Randomized Controlled Trial. *Obesity (Silver Spring)* 2019;27(1):75-86. doi: 10.1002/oby.22359 [published Online First: 2018/11/14]
44. Conroy MB, McTigue KM, Bryce CL, et al. Effect of Electronic Health Record-Based Coaching on Weight Maintenance: A Randomized Trial. *Ann Intern Med* 2019;171(11):777-84. doi: 10.7326/m18-3337 [published Online First: 2019/11/12]
